# SAFR: Enabling Fragment-Based Drug Discovery with a Synthetic Binding Pose Data Set

**DOI:** 10.1021/acs.jcim.6c00217

**Published:** 2026-04-16

**Authors:** Joan Cabot-March, Xavier Jalencas, Jordi Mestres

**Affiliations:** † Chemotargets SL, Parc Cientific de Barcelona, Baldiri Reixac 4 (TR-03), 08028 Barcelona, Catalonia, Spain; ‡ Institut de Quimica Computacional i Catalisi, 430179 Facultat de Ciencies, Universitat de Girona, Maria Aurelia Capmany 69, 17003 Girona, Catalonia, Spain

## Abstract

Fragment-Based Drug Discovery (FBDD) is a powerful strategy with a proven track record of generating potent bioactive small molecules from low-affinity chemical fragments. Computational approaches to FBDD are often limited by the availability of high-quality, structurally resolved data on fragment binding poses. To address this gap, we introduce the Structurally Augmented Fragment Repository (SAFR), a novel data set designed to support in silico FBDD. Initially, a set of 89,375 high-confident binding poses of bioactive molecules in public sources was obtained by applying a filtering protocol involving 2D ligand similarity and 3D ligand superposition against protein-bound ligand structures followed by scoring with protein–ligand docking and interaction features. Fragmentation of the bioactive ligands in their predicted binding poses resulted in a total of 818,385 fragment-protein interactions between 157,080 unique chemical fragments and environments from 1,142 distinct proteins. Of them, 270,155 are unique fragment-protein interactions, of which 237,284 (88%) are not represented in protein-bound ligands in the PDB. Case studies using SAFR for bioisosteric replacements and scaffold hopping are presented. SAFR is a useful resource to support fragment screening campaigns and hit-to-lead optimization. It is publicly available at https://zenodo.org/records/18229523 .

## Graphical abstract

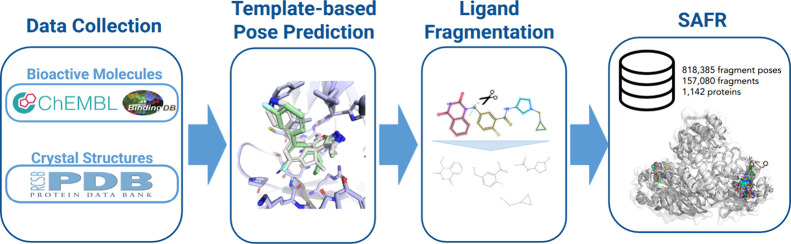

## 1 Introduction

The increasing deposition of high-resolution protein structures in the Protein Data Bank (PDB) has fundamentally changed the process of designing new drugs.^1^ The availability of such a wealth of experimentally determined X-ray structures has facilitated the application of structure-based drug discovery (SBDD)^2–4^ but it has offered also the most valuable reference set of structural data to perform high-throughput computational modeling at levels of performance never achieved before.^5,6^ Many protein structures contain interacting small molecule ligands inside binding cavities. These entries inform not only about the three-dimensional (3D) binding pose of those ligands but also about which chemical fragments fit into specific protein environments.^7,8^

Fragment-Based Drug Discovery (FBDD) relies on this structural data by screening libraries of simple, low-molecular-weight compounds rather than complex molecules.^9^ These low-affinity fragments are used as chemical seeds that can be grown or merged to generate more potent small molecules. But this optimization process depends highly on knowing the precise orientation of the fragment within the binding pocket. Unfortunately, reliably predicting these binding poses remains a significant computational bottleneck. While standard docking methods can generate many potential binding conformations, their scoring functions often fail to distinguish the correct, biologically relevant pose from incorrect ones. This pose prediction problem is a major obstacle, as an incorrect structural hypothesis can undermine the entire fragment evolution process.^10,11^

A practical way to move forward is to exploit the binding pose information existing in all available X-ray crystal structures of protein–ligand complexes in the PDB and apply the hypothesis that molecules with highly similar structures are likely to interact with a protein in a similar way. By using ligand-bound crystal structures as templates for the 3D superposition of structurally similar known bioactive ligands, we can significantly narrow down the binding mode possibilities for a new ligand that has not been crystallized, turning a difficult global search into an easier local adjustment problem. This strategy has been shown to successfully correct scoring artifacts such as the systematic misplacement of specific functional groups by standard docking algorithms.^12–15^ This notwithstanding, the method’s success remains sensitive to the selection of a representative template.

Several recent large-scale efforts, such as SAIR,^16^ GatorAffinity,^17^ or Smiles2Dock,^18^ have produced repositories of predicted protein–ligand complexes using deep learning folding models like Boltz^19,20^ or conventional docking. This work distinguishes from those previous ones by centering the focus on the interaction of small chemical fragments with local protein environments. The results are collected in a Structurally Augmented Fragment Repository (SAFR) publicly available at https://zenodo.org/records/18229523. A template-based 3D superposition workflow is used to generate high-confidence binding poses for known bioactive ligands that lack crystallographically determined structures. The ligands in their predicted binding poses are then deconstructed into their constituent fragments, resulting in a vast repository of chemical fragments interacting with protein environments. Several applications of SAFR to typical use cases of FBDD, including bioisosteric replacements and scaffold hopping, are presented.

## 2 Methods

### 2.1 Data Set Curation

The foundation of this work is a large-scale structural data set compiled from public repositories of X-ray crystal structures of proteins with a ligand bound, on one side, and small molecules with experimentally determined binding affinities for those proteins, on the other side. All data was collected based on the latest available versions as of July 2024. The entire PDB was processed to identify all proteins that had at least one structure cocrystallized with a bound small-molecule ligand. This criterion ensured a validated starting point for defining a binding pocket for each protein. The resulting structural data set, which formed the basis of our template library, comprised 615,125 ligand-protein pairs. This set represented 65,827 unique ligands (by InChIKey) and 30,063 unique proteins (by UniProt ID), sourced from 127,376 distinct PDB entries.

To compile a corresponding set of known active ligands for these proteins, we integrated data from two of the largest public bioactivity databases, namely, ChEMBL and BindingDB.^21,22^ The activity data available in these sources was mapped to the proteins identified from the PDB via their shared UniProt identifiers. All quantitative bioactivity measurements (IC_50_, EC_50_, K_i_, K_d_) were converted to a standardized logarithmic scale (pAct). Ligands with a pAct of 5.0 or greater, corresponding to a potency of 10 μM or better, were considered “active binders” and included in our data set. For entries where only qualitative annotations were available, ligands explicitly labeled as “active″ were also retained.

This process yielded two distinct sets of ligands for each protein: (1) a reference set of ligands with experimentally determined binding poses, sourced directly from the PDB, and (2) a set of known bioactive ligands, sourced from ChEMBL and BindingDB, for which no crystal structures of the corresponding protein–ligand complexes were available. This collection forms the basis for our template-based pose prediction and subsequent fragment library generation.

### 2.2 Ligand Preparation and Conformer Generation

All ligands were standardized to physiological conditions. Protonation states were assigned at pH 7.0 using the OpenBabel engine. This step ensures that ionizable groups, such as carboxylic acids and basic amines, reflect the most probable formal charges encountered in a biological environment prior to 3D embedding.

For each standardized ligand, a 3D conformer ensemble was generated using the RDKit implementation of the ETKDGv2 algorithm. To ensure high-quality starting geometries, each conformer underwent local energy minimization using the MMFF94s force field. The resulting ensemble was pruned using a heavy-atom RMSD threshold of 0.5 Å to remove redundant poses while maintaining conformational diversity.

All crystallographic water molecules, metal ions, and nonpolymeric cofactors were excluded from the template structures during the alignment and scoring phases.

### 2.3 Template Selection and Ligand Superposition

For each molecule with known affinity for a given protein but without structural information (query ligand), all ligands with crystallographically determined structure bound to that protein were collected (template ligands), and their corresponding 2D Tanimoto similarities based on radius 2 Morgan fingerprints^23^ were calculated. The three most similar template ligands were considered for further use if their 2D Tanimoto similarity exceeded 0.3. After collecting the candidate template ligands, we generated 5 diverse conformers for the query ligand. MIMIC^24^ was used to obtain the 3D superposition for each test ligand. MIMIC uses molecular field-based similarity to optimize 3D alignments between pairs of molecules accounting for all translational, rotational, and conformational degrees of freedom.^25,26^ The top three alignment poses returned by MIMIC were taken as the predicted poses for each template molecule.

### 2.4 Pose Optimization and Scoring

Next, pose refinement was performed using rDock tethered docking,^27^ where the aligned maximum common substructure (MCS) between the query and template ligands was fixed. Full configuration details and the rDock parameter block are provided in the Supporting Information (Listing S1). Several scoring parameters were calculated for each optimized pose: (i) 2D Tanimoto Similarity (*2Dsim*) captures the 2D structure similarity between the query and template ligands; (ii) cosine-like field-based 3D molecular field similarity (*3Dsim*) reflects the 3D similarity of the optimally superimposed steric and electrostatic fields; (iii) QuickVina2 Score (*IntDock*)^28^ assesses the goodness of fit of the optimized ligand pose inside the protein cavity. This was calculated using the --score_only flag with default parameters to evaluate the SAFR-generated pose without further stochastic search; and (iv) Interaction Similarity (*IntSim*) that returns an estimated affinity based on Extended Connectivity Interaction Features (ECIFs).^29^ All four parameters were normalized to a range of 0 to 1. A global confidence score (CS) was then computed as *CS* = α · 2*Dsim* + β · 3*Dsim* + γ · *IntDock* + δ · *IntSim*, where α, β, γ, and δ represent the weights assigned to each metric with values in the range [0,1]. All weights were optimized for the PDBbind Core set^30^ by a weighted linear combination through an exhaustive grid search that maximizes the F1 score to ensure a robust balance between precision and recall in identifying successful docking poses (RMSD < 2 Å). A perturbation study is included in Figure S1. The final optimal values are α: 0.027, β: 0.324, γ: 0.378, and δ: 0.270.

### 2.5 Computational Cost

The complete end-to-end process encompassing template querying, 3D conformer generation, structural alignment, pose optimization, and final scoring required a median runtime of 94.6 s per template, with an interquartile range of 47.1 to 203.3 s, executed on a single Intel Xeon Gold 6230R (2.10 GHz) core. Because the optimization protocol involves exploring conformational degrees of freedom, the computational cost depends on ligand flexibility, scaling with the number of rotatable bonds **(** Figure S2 **)**.

### 2.6 Validation Protocol

To assess the accuracy and reliability of our template-based strategy, we performed a validation study using the PDBbind data set (v2020). From this benchmark data set, several filters were applied to curate a final subset suitable for our protocol. First, peptides and polysaccharides were removed. Furthermore, to ensure that a valid template could always be selected, we excluded complexes where the protein had only a single crystallographic ligand present in the refined set, as no alternative template would be available for the cross-validation procedure. This resulted in a final validation set composed of 8,011 entries used for the analysis.

The validation procedure was executed for each complex in this final set as follows. First, the ligand of interest was temporarily treated as a query ligand, with its experimentally determined pose serving as the “ground truth” reference for the prediction. To this end, the query ligand itself was excluded from the pool of potential templates for that specific protein. Once the pool of templates was collected, the pose prediction pipeline was executed and the pose with the higher confidence score retained.

The accuracy of the final predicted pose was quantified by calculating the heavy-atom Root Mean Square Deviation (RMSD) relative to the original crystallographic pose. Prior to the RMSD calculation, the protein backbones of the predicted and reference complexes were superimposed to establish a common frame of reference and ensure that the measurement reflects only the ligand’s conformational and positional accuracy. Following established conventions in the field for pose prediction success, a predicted pose was classified as successful if the resulting RMSD was less than 2.0 Å.

To further evaluate the effect of binding-mode multiplicity on pose prediction performance, we quantified the number of distinct binding pockets for each target. For a given protein, all available experimental structures were structurally aligned to the reference PDB entry. The crystallized ligands from these aligned structures were then extracted and spatially clustered to define discrete, physically separate binding sites.

### 2.7 Fragment Library Generation

Following the validation of our template-based pose generation, we proceeded to construct a structurally annotated fragment library. The starting pool of modeled ligands was first filtered based on the confidence score of their predicted poses. Only ligands with a confidence score of 0.6 or greater were selected for the subsequent fragment deconstruction process, ensuring that the resulting fragments are derived only from the most reliable binding hypotheses.

The fragmentation of this high-confidence set was performed using the Breaking of Retrosynthetically Interesting Chemical Substructures (BRICS) algorithm.^31^ BRICS was chosen because it severs bonds that are considered synthetically tractable, ensuring that the resulting fragments are chemically reasonable and relevant for potential follow-up chemistry. A key aspect of our protocol was an exhaustive decomposition strategy. Instead of retaining only the smallest possible fragments from the BRICS cleavage, we generated and collected every possible substructure that could be created by the disconnection rules. This approach allowed us to capture a richer variety of fragments, including larger core scaffolds and linkers embedded within the parent active molecules.

This initial set of fragments was further refined based on a set of fragment-like properties derived from an analysis of 131 fragments that were successfully developed into lead compounds.^32^ A generated fragment was retained in the final library only if it simultaneously satisfied all of the following criteria: (i) molecular weight (MW) ≤ 300 Da, (ii) number of hydrogen bond donors (HBD) ≤ 3; (iii) number of hydrogen bond acceptors (HBA) ≤ 6; (iv) number of rings (NR) between 1 and 4 (both inclusive); and (v) number of rotatable bonds (NRB) ≤ 5. The result is a collection of chemical fragments, each paired with high-confidence 3D coordinates defining its orientation and interactions within a specific protein environment.

A schematic representation of the entire pipeline to generate SAFR from PDB and ChEMBL is illustrated in Figure 1.

**Figure 1:**
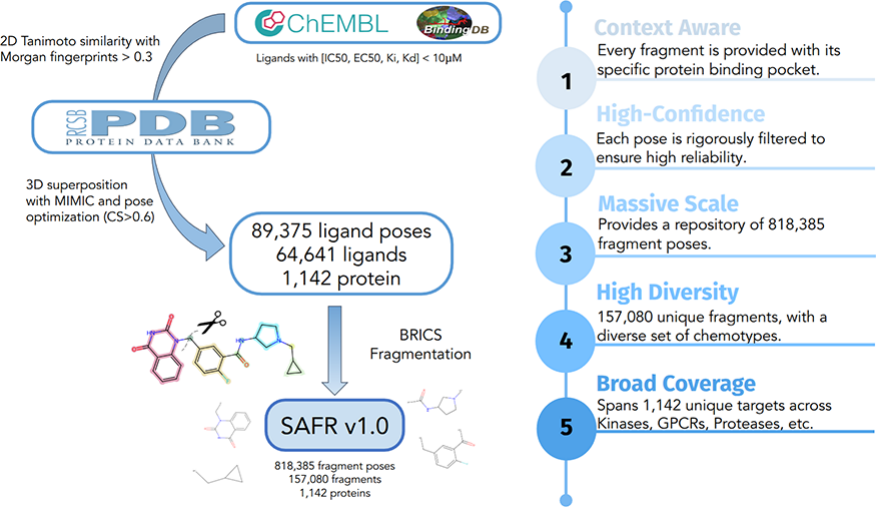
**Schematic representation of the SAFR Repository.** Bioactivity data is collected from ChEMBL and BindingDB and the template docking protocol is applied to all Active compounds, those with High confidence (CS > 0.6) are then fragmented via the BRICS algorithm to create the 3D annotated fragment repository.

## 3 Results and Discussion

### 3.1 Validation of Pose Prediction

The performance of our template-based pose generation was assessed using a validation set derived from the PDBbind v2020 data set. A valid binding pose (RMSD ≤ 2.0 Å) was predicted for 68.6 ± 0.3% of the query ligands. This result reinforces the core hypothesis that using structurally similar templates can reliably guide pose prediction for a broad range of molecules. Furthermore, when filtering for predictions with a Confidence Score >0.6, the confidence threshold used to select ligands for the final fragment library, the percentage of valid binding poses went up to 83.9 ± 0.5%. This performance compares favorably to redocking with rDock (44.6%) and Boltz-2 (79.1%). Notably, the Boltz-2 success rate likely benefits from data leakage, as a substantial portion of the systems set were included in its original training data.

The cumulative percentage of ligands predicted within a given RMSD threshold is shown in Figure 2A. As can be observed, for over 50% of the ligands a binding pose with RMSD less than 1.0 Å is obtained and a significant enrichment of binding poses with RMSD values below 2.0 Å is achieved when a confidence score threshold (0.6) is imposed. In addition, an analysis of binding-mode multiplicity revealed no significant correlation between the number of distinct binding sites per target and pose prediction accuracy (Figure S3). The dependency of binding pose success on template similarity was assessed by analyzing the distribution of median RMSD values (out of 5 runs) across similarity ranges. Interestingly, at the 2D level, most median RMSD values are kept below 2.0 Å, even at low 2D Tanimoto similarities (Figure 2B). In contrast, 3D molecular field similarities are more sensible than 2D Tanimoto similarity and low values (below 0.5) tend to return binding poses with RMSD values above 2.0 Å (Figure 2C). Overall, binding pose prediction accuracy exhibits a substantially stronger dependence on 3D spatial superpositions than on 2D structural overlap.

**Figure 2:**
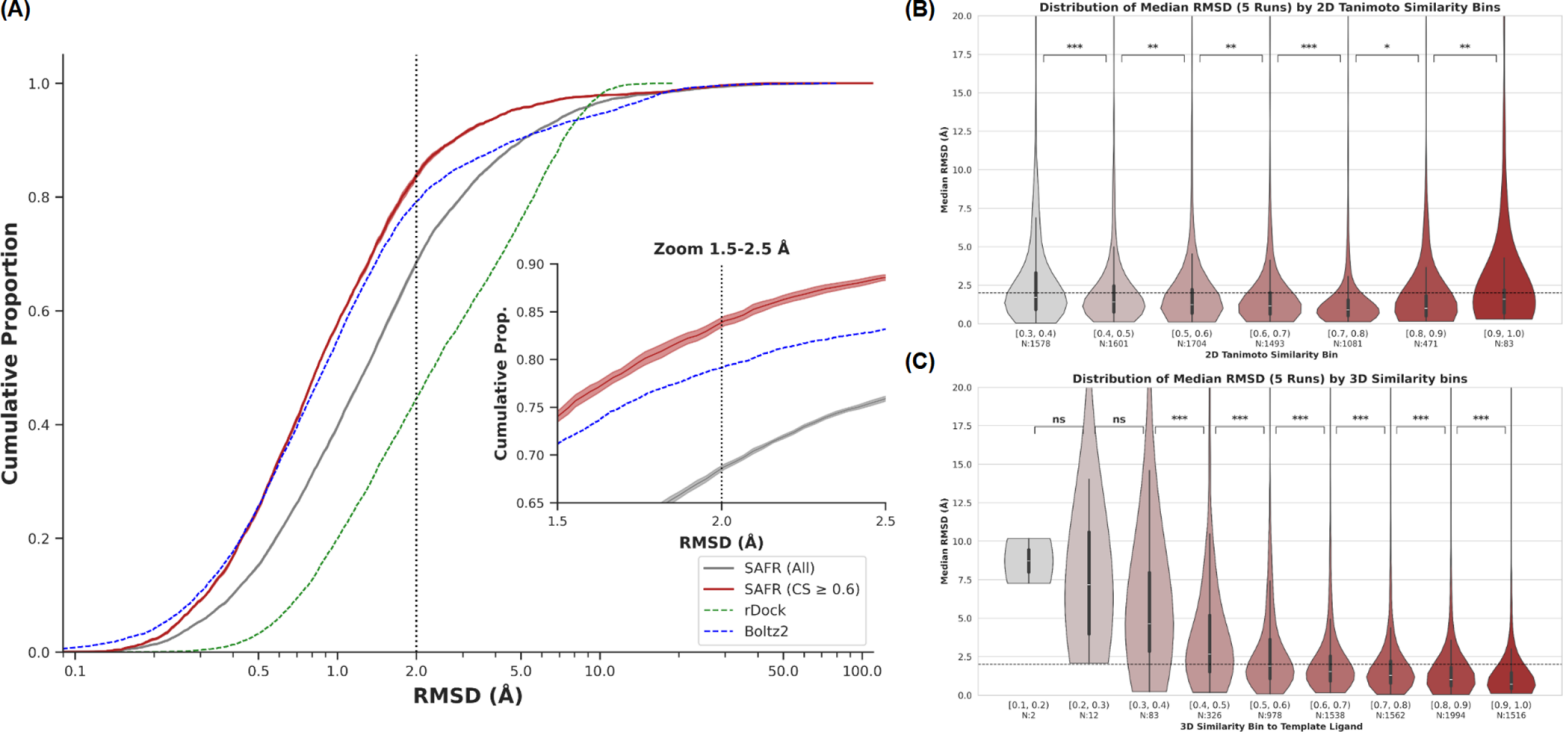
**Pose Prediction Performance. (A)** Cumulative percentage of ligands (*y*-axis) successfully predicted within a given heavy-atom RMSD (*x*-axis, logarithmic scale) from their crystallographic pose. The gray curve represents the performance across all ligands in the validation set for the median 5 runs alongside the standard deviation, whereas the red curve illustrates the accuracy for the high-confidence subset (Confidence Score >0.6). For benchmarking, state-of-the-art deep learning (Boltz-2, blue dashed line) and classical docking (rDock, green dashed line) baselines are included. The vertical dotted line marks the standard 2.0 Å success threshold. An inset has been added to be able to appreciate the low deviation along 5 runs. (B) Distribution of the median prediction RMSD binned by 2D Tanimoto similarity to the template ligand. (C) Distribution of the median prediction RMSD binned by 3D template similarity.

A closer look at individual cases provides insight into the strengths and limitations of the strategy adopted (Figure 3). For example, the binding pose of ligand from PDBbind entry 5YG3 was modeled using the ligand from 4TN4 as a template, yielding an exceptionally good prediction (RMSD = 0.61 Å). In this instance, a good binding pose was obtained despite a relatively low Tanimoto 2D similarity of 0.47 between the two molecules. Conversely, the binding pose of ligand from PDBbind entry 3R8I was modeled using a highly similar template from 2I4P (Tanimoto 2D similarity of 0.85). However, in this case the pose generated was in poor agreement with the crystallographic data (RMSD = 3.55 Å). Inspection of the respective crystal structures reveals that although the two ligands are chemically similar, they bind in slightly different regions of the same pocket. Specifically, a long aliphatic chain present in both molecules occupies a distinctly different region of the binding site in each complex. The template-based alignment correctly placed the interacting carboxyl group but it was unable to foresee the significant conformational rearrangement of the flexible chain and rest of the molecule. Such cases indicate that while the strategy is effective, its predictive ability can be limited when structurally similar ligands adopt different binding modes, a known and long recognized challenge for any ligand-based and structure-based method. Additional examples of potential sources for failure of template-based approaches involving highly flexible ligands, inverted binding modes between similar ligands and/or high pocket plasticity are shown in Figure S4.

**Figure 3:**
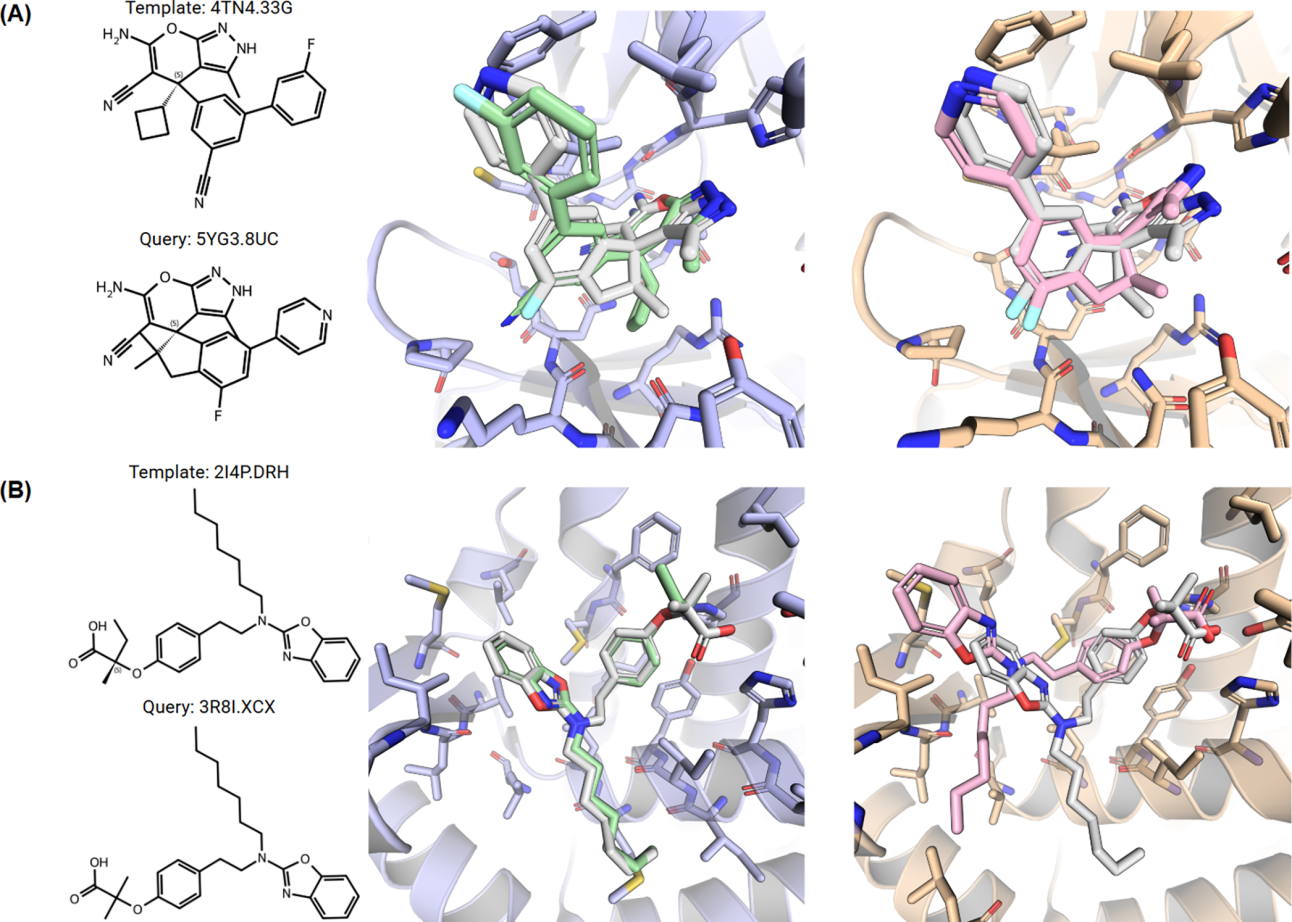
**Examples of Pose Prediction Outcomes.** The figure compares a successful and an unsuccessful prediction. In all 3D views, the predicted pose is white, the template pose is green and the crystal pose is pink. (A) A successful prediction (RMSD = 0.61 Å) for the 5YG3 ligand, which was correctly modeled using the dissimilar 4TN4 ligand (Tanimoto = 0.47) as a template. (B) An unsuccessful prediction (RMSD = 3.55 Å) for the 3R8I ligand. Despite high similarity to its 2I4P template (Tanimoto = 0.85), the pose was incorrectly predicted due to a different conformation of a flexible chain.

### 3.2 Chemical Plausibility of Predicted Poses

Beyond positional accuracy, the chemical plausibility of our predicted poses was evaluated using PoseBusters,^33^ a tool that assesses the validity of the modeled ligand-bound conformation according to several metrics, such as bond lengths, angles, internal energies, and steric clashes, among others. Three distinct sets were analyzed: (i) the original bound ligands in the PDBbind set, (ii) all poses generated using our template-based protocol, and (iii) the high-confidence subset (Confidence Score >0.6). The results are summarized in Table 1. The original crystal ligands themselves exhibited a baseline failure rate of 1.71%. In contrast, the set of all our predicted poses had a failure rate of 5.16%. However, filtering all poses by confidence score significantly reduced the failure rate down to 2.59%, a value close to the experimental baseline (1.71%) and slightly below the 3% reported in SAIR.^16^ An analysis of the failures within the high-confidence set revealed that they were primarily due to high internal energy (62.76% of failures) and internal steric clashes (44.68% of failures), suggesting minor residual strain not fully resolved by the minimization protocol.

**Table 1:** PoseBusters Chemical Plausibility Analysis

				**Failure Breakdown (% of Fails)**				
**Pose Set**	**N**	**Pass Rate (%)**	**Overall Failure Rate (%)**	**Internal Energy**	**Internal Steric Clashes**	**Aromatic Ring Flatness**	**Bond Angles**	**Bond Lengths**
Crystal Ligands (PDBbind)	8,011	98.29	1.71	37.22	45.25	3.65	7.30	17.52
All Predicted Poses	8,011	94.84	5.16	72.86	34.96	0	0	0
High-Confidence Predicted Poses(CS > 0.6)	3,622	97.41	2.59	62.76	44.68	0	0	0

### 3.3 Prospective Validation against Newly Released Crystal Structures

During the preparation of this manuscript, X-ray crystal structures of 14 ligand-protein complexes were published for known bioactive ligands that had no experimental structure available at the time we generated binding poses. Following the public release of their crystal structures in the PDB after January first, 2025, we compared our pre-existing predictions to the X-ray bound poses. All 14 poses generated prior to the PDB release successfully captured the experimental binding modes, with RMSD values under 2.0 Å (Table S1).

At a larger scale, the binding poses obtained in this work were compared with those deposited recently in the SAIR data set.^16^ For each overlapping protein–ligand pair, we selected the single highest-scoring pose from the five hypotheses provided by SAIR. A prefiltering step was applied to ensure a meaningful comparison: since the SAIR approach involves cofolding, the protein structures from both predictions were first aligned, and only pairs with a protein–protein RMSD below 2.0 Å were retained for the subsequent ligand RMSD analysis. This protocol gave us a set of 59,798 comparable poses. Within this set, 41.98% of ligand poses agreed within an RMSD of 2.0 Å and a median RMSD of 2.43 Å. A closer agreement was obtained when focusing on the subset of high-confidence predictions from both methods. By applying our confidence threshold of >0.6 and the >0.8 threshold used in the original SAIR publication, a total of 14,882 poses were left. In this high-confidence subset, the concordance rate increased to 63.17% and the median RMSD improved to 1.52 Å (Figure 4). These results provide mutual support for the binding poses generated independently for the corresponding high-confidence subsets.

**Figure 4:**
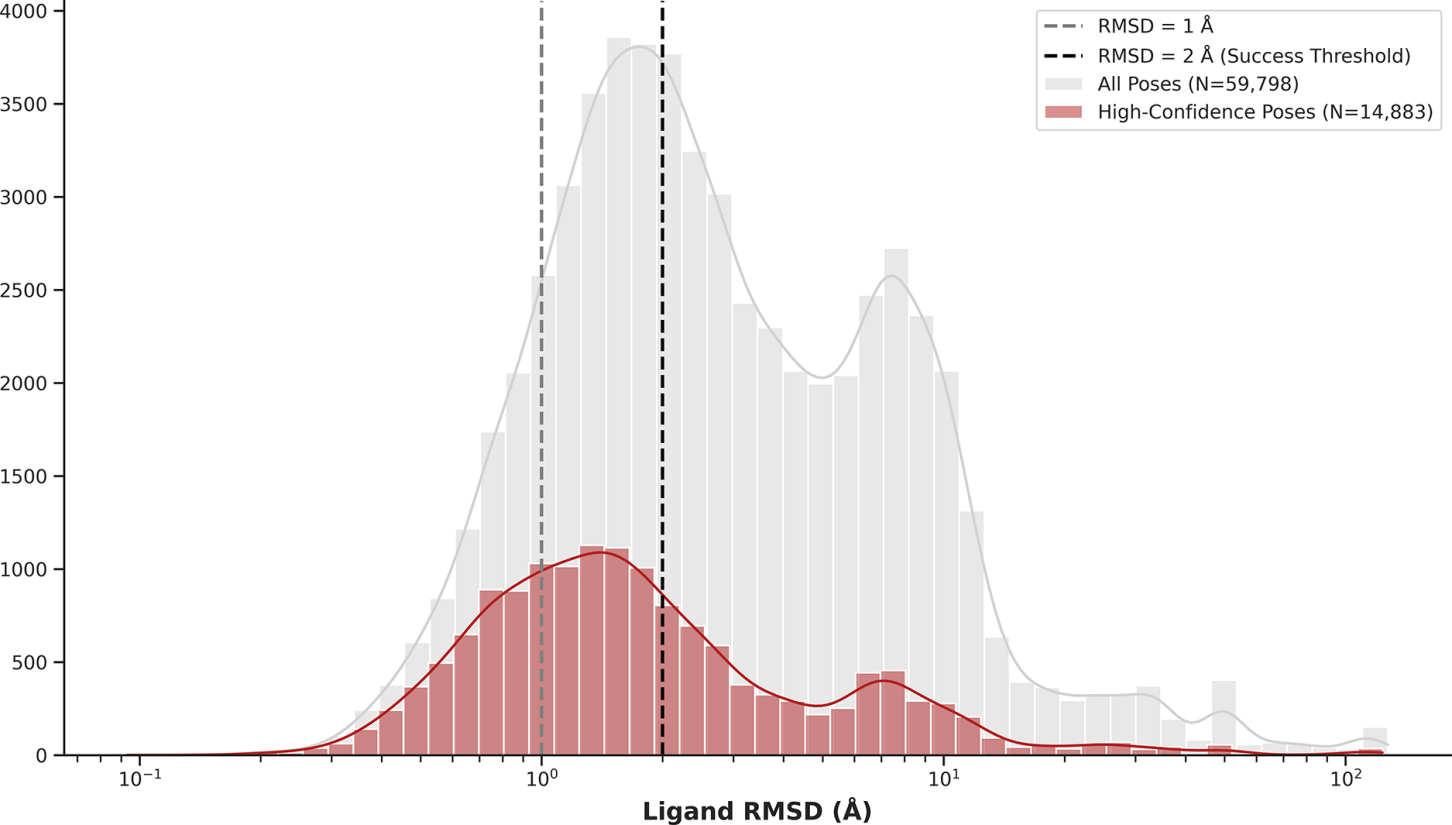
**Concordance of Predicted Poses with the SAIR Data set.** The figure shows the distribution of RMSD values between poses predicted in this work and those from the SAIR data set. The gray distribution represents all 59,798 comparable pairs (median RMSD = 2.43 Å), while the overlaid red distribution represents the 14,883 pairs where both methods reported high confidence scores (median RMSD = 1.52 Å). The significant shift toward lower RMSD in the high-confidence subset highlights the strong agreement between the two methodologies for their most reliable predictions.

### 3.4 Fragment Library Analysis

The generation of the ’predicted fragments’ database, defined here as substructures generated with the fragmentation algorithm from bioactive ligands with known target activity, depends on their high confidence predicted binding pose. The fragment library derived from high-confidence ligand poses will be compared against a reference library created by applying the identical fragmentation and filtering protocol to experimentally determined ligand poses (“crystal fragments”) from protein–ligand complexes in the PDB. To generate a Structurally Augmented Fragment Repository (SAFR), a total of 422,183 known active ligands sourced from ChEMBL and BindingDB were processed and fragmented into 7,502,153 fragments, of which 644,448 are unique. Applying our confidence score filter (CS > 0.6) yielded 89,375 high-confidence predicted ligand poses. This high-confidence set represents 64,641 unique ligands placed into the binding cavities of 1,142 distinct protein targets, using templates derived from 7,690 unique PDB entries. To assess the potential for systematic structural bias, we analyzed the distribution of template reuse across the data set. On average, each template was used to model 7.9 ligands. However, this distribution is highly skewed (Figure S5). The median template reuse is 3 ligands per template, indicating that most templates are not overrepresented. While a small subset of highly promiscuous templates does generate larger clusters of fragments, the overall set of 8,806 templates ensures high structural diversity and mitigates the risk of propagating systemic template bias into the wider SAFR fragment space.

Fragmentation of the 89,375 ligand poses resulted in a library of 818,385 predicted fragments, of which 157,080 are unique. This is more diverse than the set of crystal fragments that contains 107,756 unique fragments in its approximately 2.1 million fragments. A total of 27,492 fragments are common to the unique sets of predicted and crystal fragments, meaning that 82.5% of SAFR represents novel fragment matter within specific binding environments. The properties of both predicted and crystal fragments show nearly identical distributions (Figures 5A and S6A, and Tables S2 and S3). The relationship between size (MW) and hydrophobicity (clogP) and the number of rule of three (Ro3) violations of the fragments in SAFR (Figure 5B,C) are also comparable to those obtained for crystal fragments (Figure S6B,C).

**Figure 5:**
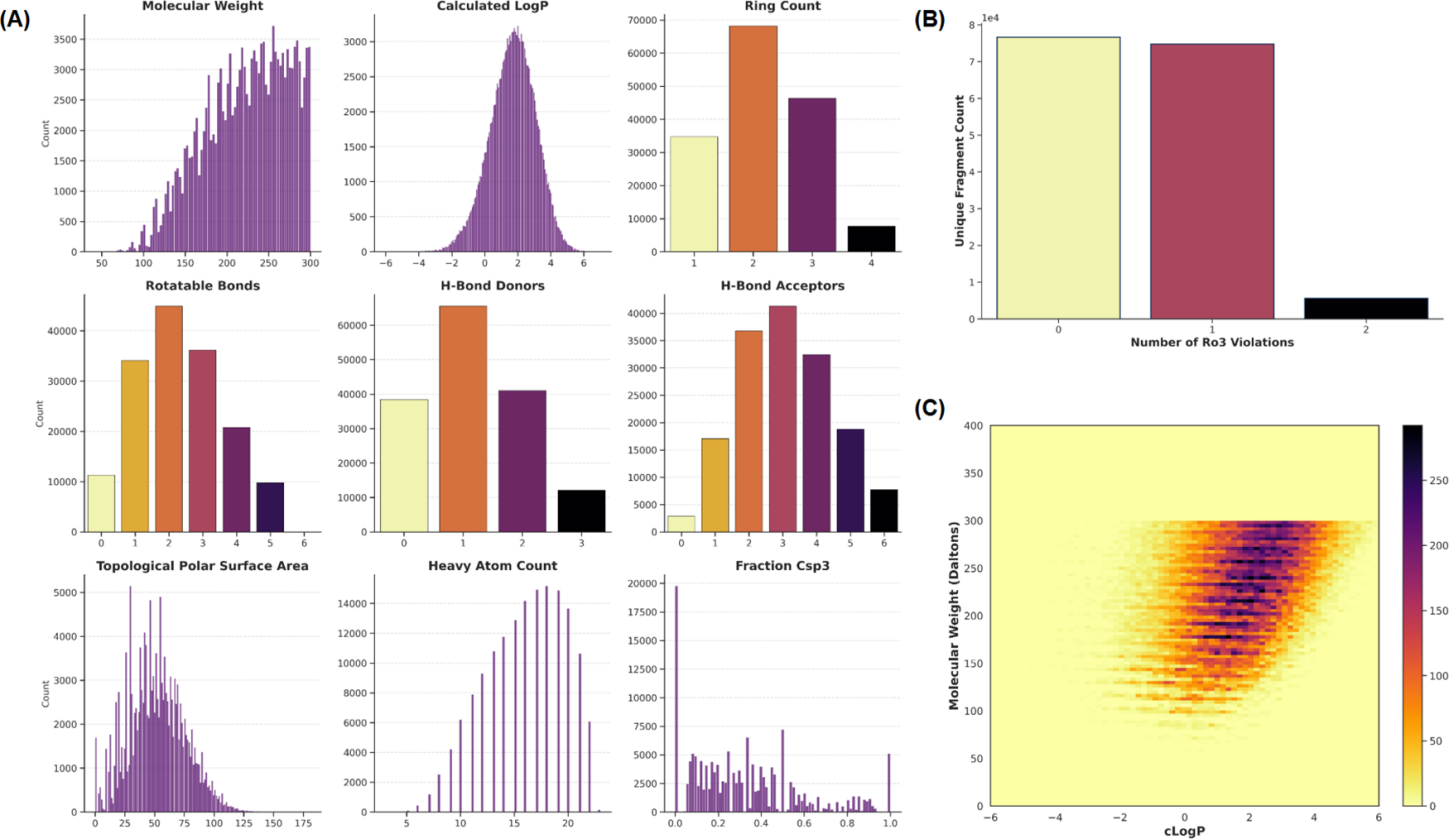
**Physicochemical Property Distributions of Predicted Fragment Library. (A):** Grid of distribution plots for nine key properties: Molecular Weight, cLogP, Ring Count, Rotatable Bonds, H-Bond Donors and Acceptors, TPSA, Heavy Atom Count, and Fraction Csp3. The property distributions show the high quality of the computationally generated set. **(B):** A 2D density heatmap illustrating the distribution of fragments in the chemical space defined by Molecular Weight and cLogP, confirming that our library is concentrated in the desirable region for FBDD. **(C):** A bar plot comparing Rule of Three (Ro3) compliance showing that our library exhibits fragment-like character, with the majority of compounds having zero or one violation.

The structural novelty was assessed through a comparative frequency analysis of predicted and crystal fragments (Figure 6). When comparing the respective fragment occurrence frequencies (Figure 6A), we observe a specific subset of chemical structures that appear with high frequency in the crystal fragments set but are rare or absent in the predicted fragments set. Structural inspection reveals that they are predominantly saccharide-like rings and carbohydrate derivatives, reflecting the high prevalence of crystallographic sugar additives, cofactors, and glycosylated proteins in the PDB, while being less represented in public databases of bioactive molecules. Interestingly, the overall population of the scatterplot is asymmetric, with a higher number of unique fragments appearing more frequently in the predicted fragments set (59.53%). This skew indicates that the fragments coming from bioactive small molecules are a good source of fragment diversity, complementing what is currently captured by crystal structures alone. The list of most frequent fragments unique to the set of predicted fragments is also provided (Figure 6B). Unlike the crystal set, dominated by fragments containing simple rings, the unique fragments in the predicted set exhibit a slightly higher degree of structural complexity. These fragments feature distinct substitution patterns and fused ring systems that go beyond simple monocyclic sugar derivatives, offering larger, more developed starting points for hit generation and library design. In order to check if the added data points represent real diversity or simple modifications of already represented fragments, we analyzed their Murcko scaffolds (Figure 6C,D). The analysis shows that in terms of scaffolds there is also a higher number of unique fragments appearing more frequently in the predicted fragments set (59.62%), with up to 16,517 exclusive scaffolds. Additionally, SAFR was further evaluated through a head-to-head comparison with an equivalent fragmentation of the SAIR data set, revealing a high degree of complementarity between the two resources. Rather than showing redundant overlap, SAFR significantly expands the reachable chemical space by providing structural data for 925 protein targets that are absent in the SAIR repository. Within these target environments, SAFR introduces 57,350 fragment poses belonging to 3,889 unique Murcko scaffolds.

**Figure 6:**
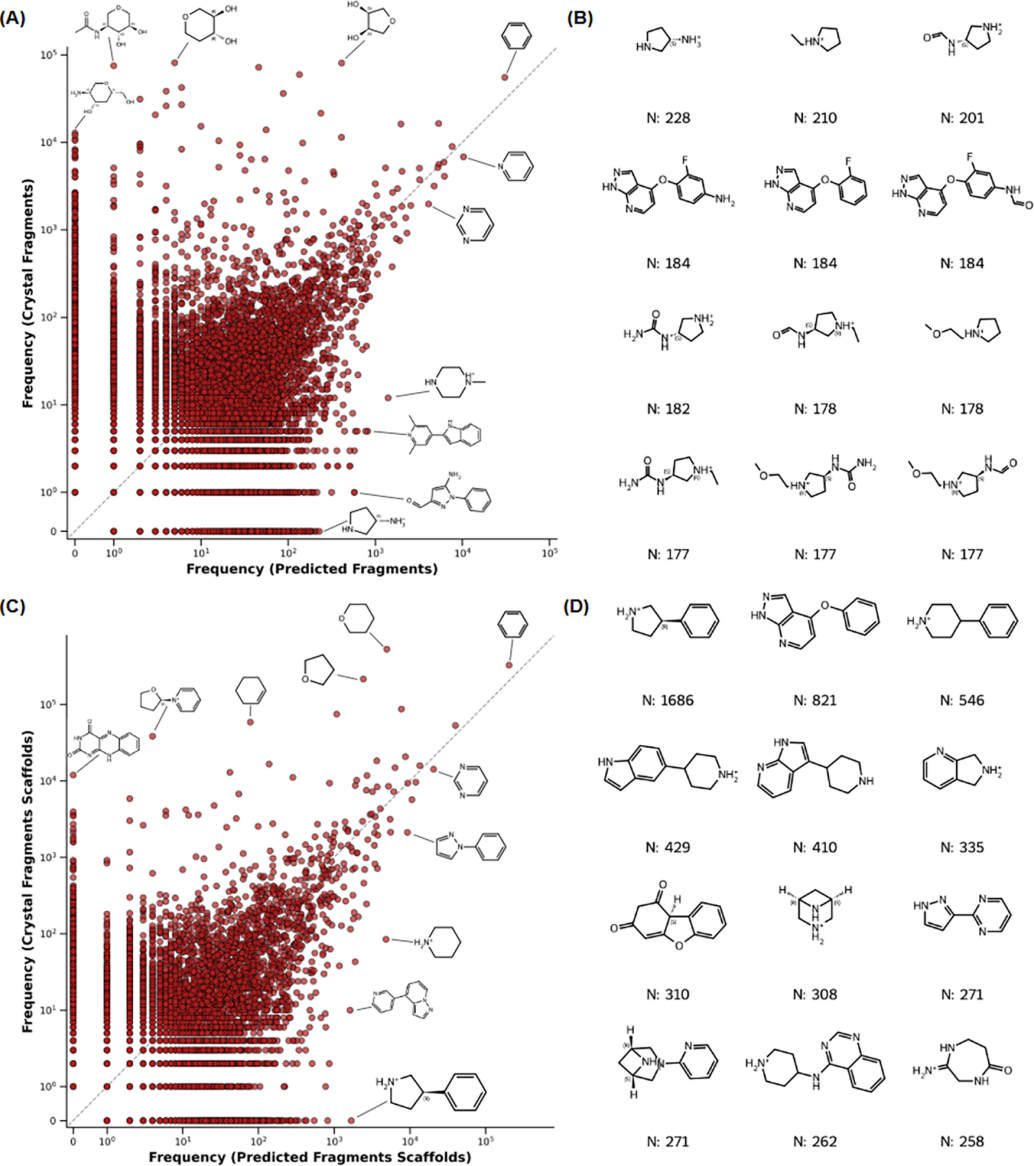
**Frequency analysis of Crystal and Predicted fragment libraries. (A):** Scatter plot showing the correlation of fragment frequencies between the two data sets (logarithmic scale), with key structures highlighted. **(B):** Chemical structures of the top 12 most abundant fragments of the Predicted Fragments set and not present in the Crystal library. **(C):** Scatter plot showing the correlation of fragment Murcko scaffolds frequencies between the two data sets (logarithmic scale), with key scaffolds highlighted. **(D):** Chemical structures of the top 12 most abundant fragment scaffolds of the Predicted Fragments set and not present in the Crystal library.

### 3.5 Expansion of Structurally Annotated Protein Families

To quantify the expansion of structural coverage provided by the predicted fragments, the density of structural data available for each protein in the crystal fragments set (21,535 proteins) was compared against that for the predicted fragments set (1,142 proteins). As illustrated in Figure 7A, many proteins fall below the diagonal, indicating an enrichment in the number of predicted fragments per protein. This also implies a wider fragment coverage of protein binding environments, revealing specific fragment-subpocket relationships that are absent or rare in the currently available structures of protein–ligand complexes in the PDB. The nuclear receptor RORγ (UniProt Accession number: P51449) is an example of a protein with significantly enriched predicted fragments in high-confidence poses (Figure 7B).

**Figure 7:**
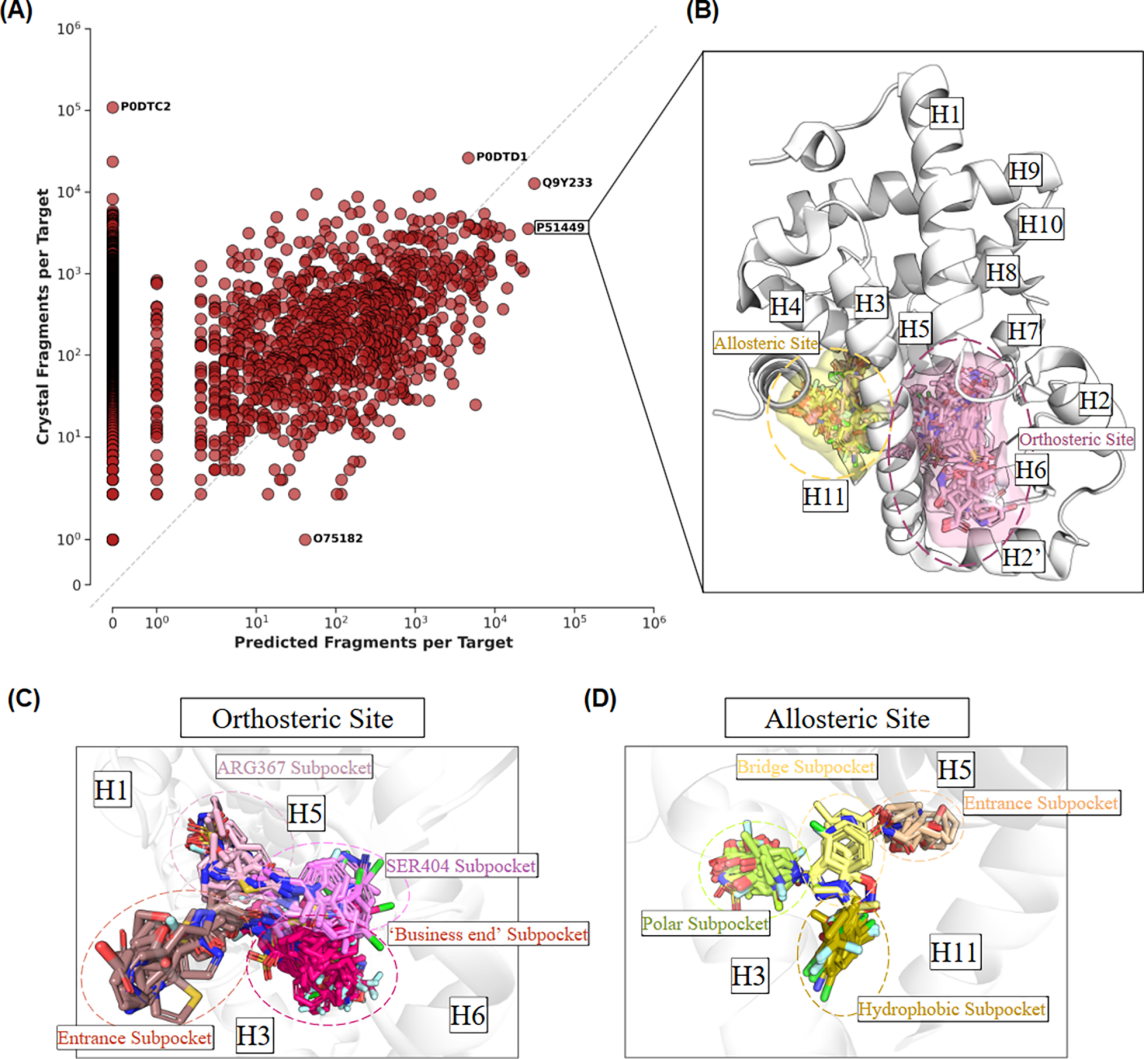
**Frequency analysis of targets represented in the Crystal and Predicted fragment libraries. (A):** Scatter plot comparing the number of unique fragments per target in the Crystal Fragments set (*y*-axis) versus the Predicted Fragments set (*x*-axis) on a logarithmic scale. Points below the diagonal represent targets where the predicted library provides greater structural coverage. Representative targets are labeled by their UniProt ID. **(B):** A structural overview of the Nuclear Receptor ROR-gamma (P51449), a target highly enriched in the predicted set. Thousands of predicted fragments are shown clustered into the orthosteric site (pink) and the allosteric site (yellow). **(C):** Detailed view of the orthosteric site, where fragments act as probes to define specific subregions: the Entrance (brown), “Business end” (magenta), SER404 neighborhood (pink), and ARG367 neighborhood (light pink). **(D):** Detailed view of the allosteric site, showing fragments occupying the Entrance (wheat), Hydrophobic (olive), Polar (lime), and Bridge (pale yellow) subpockets.

The spatial distribution of these predicted fragments clusters into distinct, biologically relevant subregions within both the orthosteric and allosteric sites of RORγ when all predictions are overlaid on PDB entry 5APK. In the orthosteric pocket (Figure 7C), predicted fragments occupy specific interaction zones, including the canonical business end, the ARG367 and SER404 subpockets, and the solvent-exposed entrance.^34^ In the allosteric pocket (Figure 7D), depending on their features, predicted interacting fragments organize in the different hydrophobic, polar, and bridging subpockets.^35^ By analyzing which fragments populate specific subpockets, we can gain immediate insights into the pharmacophoric features required to engage specific residues (e.g., SER404 or ARG367) and recognize potential bioisosteric replacements within different regions of the binding site.

Overall, the predicted fragments in SAFR contribute to a total of 1,208,133 unique pairs of interacting chemical fragments and protein environments that are not present in crystal structures of protein–ligand complexes, of which 237,284 contain predicted fragments present in high-confidence ligand poses. This represents a significant expansion of the structurally annotated fragment-protein landscape. When the proteins for the novel pairs are classified into major functional families (Figure 8), a biased distribution toward well-established therapeutic target classes is observed. Kinases are the most represented family, followed by peptidases/proteases, reductases, nuclear receptors and G protein-coupled receptors (GPCRs).

**Figure 8:**
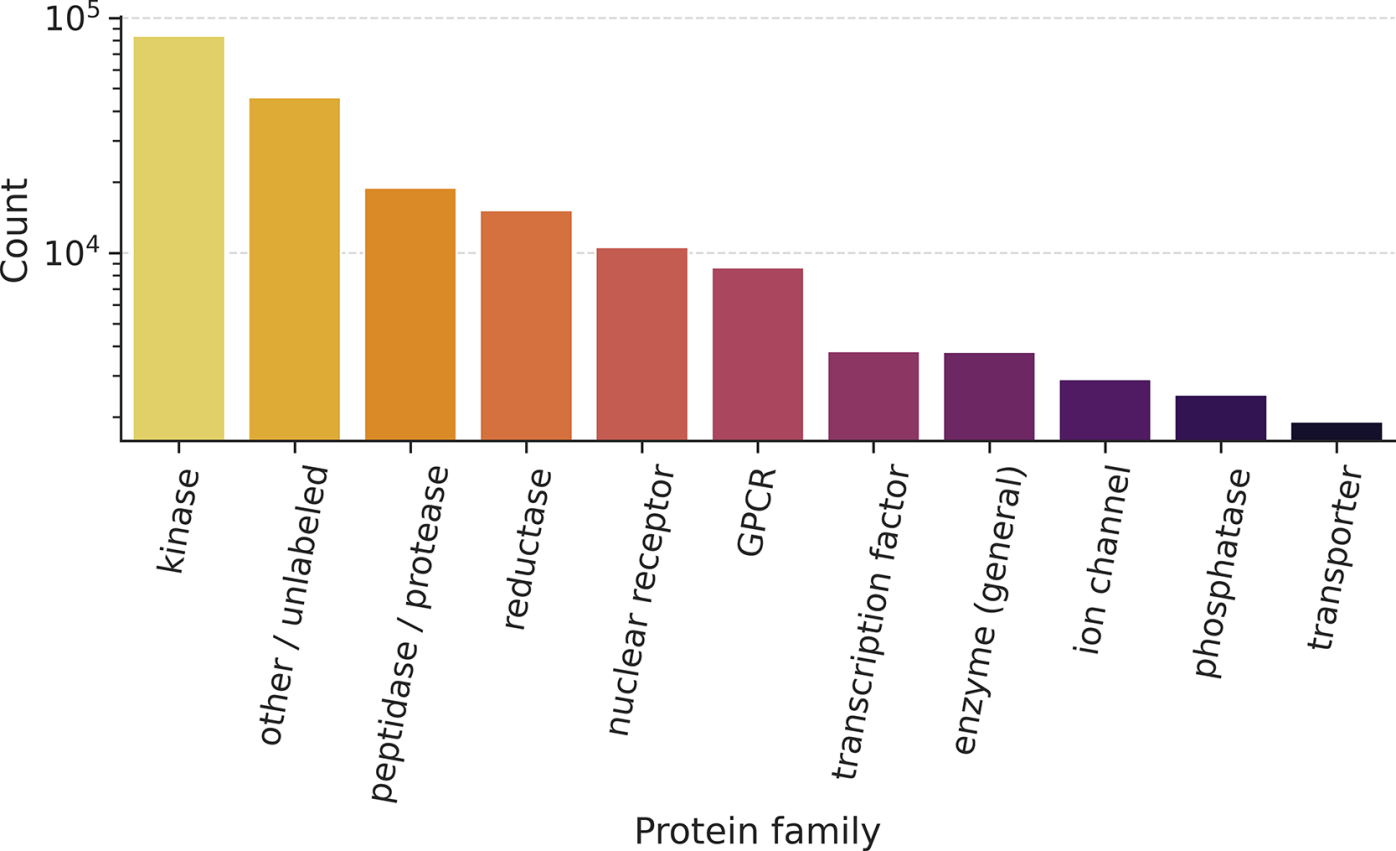
Protein Family Distribution of Fragment-Target Pairs. The bar chart shows the distribution of the 201,595 novel fragment-protein pairs across major protein families. The data distribution shows the library’s enrichment in therapeutically critical classes, with kinases representing the largest family.

Additionally, we performed an enrichment analysis to identify Murcko scaffolds in fragments that were most frequently associated with drug target families within our set of fragment-protein pairs. We focused on kinases and GPCRs as representative examples, with an analysis of other families provided in Figures S7–S13. The results recover well-known privileged structures for each protein family. For the kinase family, the analysis identified an enrichment of scaffolds that are analogues of the adenine core present in many kinase inhibitors designed to target the ATP-binding site.^36^ We can see how the most common fragment scaffold unique to kinases consistently binds the hinge region in the same way across different kinases (bottom inset in Figure 9A). In contrast, scaffolds containing biphenyl and related rigid, aromatic motifs^37^ as well as a basic amine containing rings, were more common in GPCRs.^38^ The top inset of Figure 9A shows the most common scaffold unique to GPCRs. Figures 9B and 9C contain the structures of the top nine fragment scaffolds that are unique to GPCRs and kinases, respectively.

**Figure 9:**
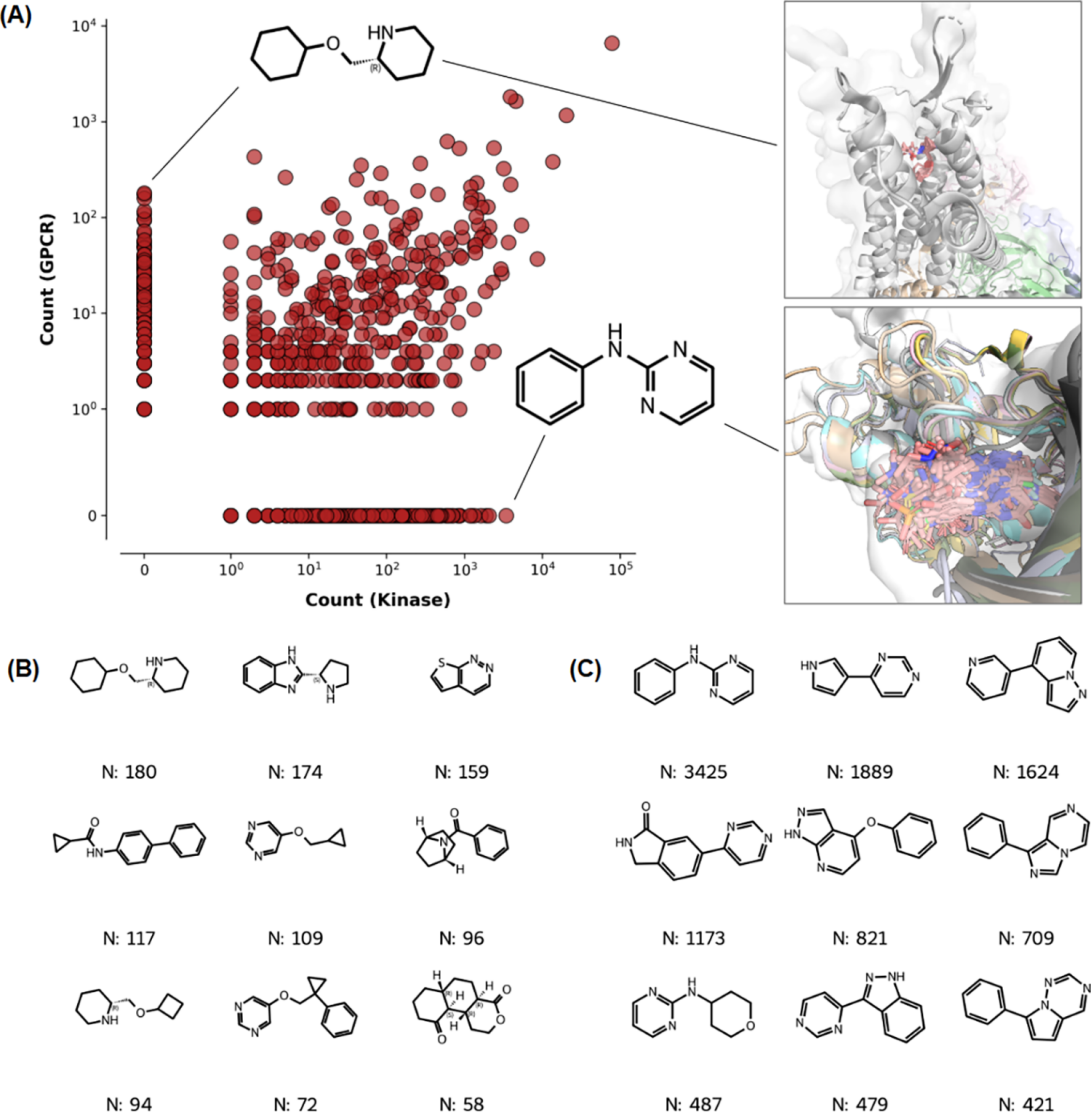
Enriched Scaffolds for Kinase and GPCR Target Families. (A): Scatterplot of Frequencies for fragments’ Murcko scaffolds appearing in Kinases (*x*-axis) vs GPCRs (*y*-axis) in logarithmic scale with insets displaying the binding predictions for the top scaffolds that only appear in each class. (B): Top 9 most appearing scaffolds found only in GPCRs. (C): Top 9 most appearing scaffolds found only in Kinases. N: count of fragment instances containing the scaffold.

To provide further validation of SAFR content at the fragment level, we performed a prospective-style validation using the SARS-CoV-2 Main Protease (Mpro), a well-characterized system with extensive crystallographic fragment screening data (PDB group deposition IDs: G_1002152, G_1002155, G_1002163, G_1002232, G_1002272, G_1002326). Our SAFR ensemble, composed of 4034 fragments, was benchmarked against 611 experimental Mpro structures, some of which were released after our data set collection.

As shown in Figure 10A, SAFR successfully identifies and populates the major binding regions of the Mpro protomer, including the catalytic dyad (Cys145/His41) and the S1/S2 subsites. A detailed comparison of specific SAFR fragments against experimental hits released after the completion of SAFR (Figure 10B) demonstrates high spatial and chemical fidelity. For example, SAFR accurately predicted the binding modes for chemical structures later confirmed in PDB entries such as 7HUC and 7I1F, even when the fragments were identified as bioisosteric mimetics rather than identical molecules.

**Figure 10:**
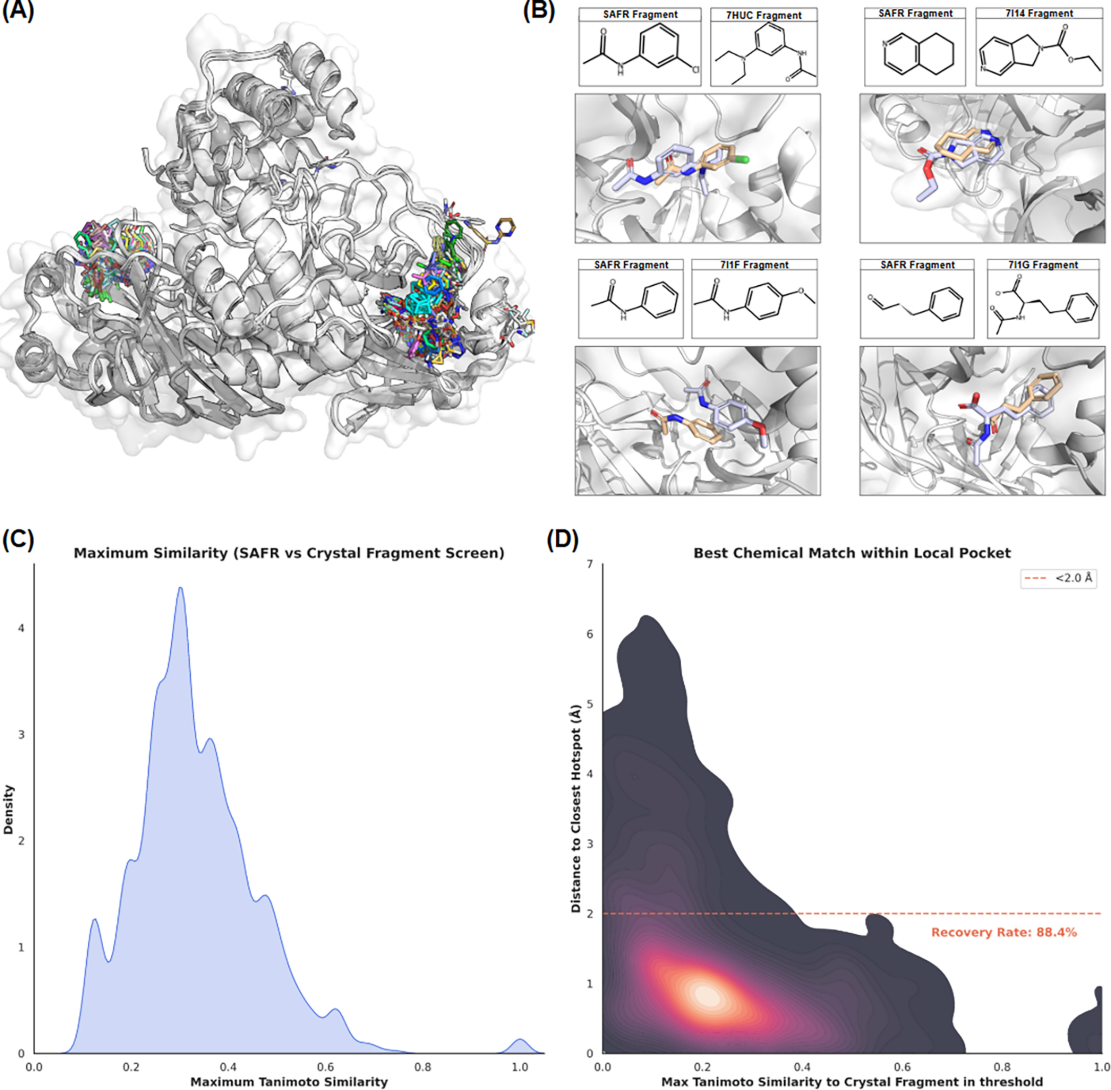
**Fragment-Level Validation of SAFR on SARS-CoV-2 Main Protease (Mpro). (A)** Structural ensemble of SAFR-generated fragments mapped onto the Mpro protomer. The distribution demonstrates comprehensive occupancy of the primary binding site. (B) Representative examples of prospective fragment recovery. SAFR-predicted poses (wheat) are compared against experimental crystallographic hits (lavender) released in 2025 (PDB IDs: 7HUC, 7I14, 7I1F, 7I1G). The predictions successfully identify similar groups with similar binding poses. (C) Maximum Tanimoto similarity between the SAFR library and the fragments from the 611 structures from the fragment screenings. The distribution highlights a diverse library with some chemical overlap with experimental hits. (D) Density distribution correlating spatial accuracy (minimum centroid-centroid distance) with chemical similarity. The high-density “Success Zone” (bottom) indicates that 88.4% of SAFR-predicted fragments are positioned within 2.0 Å of an experimentally verified crystallographic hotspot. The presence of low-distance, low-similarity points demonstrate the tool’s capacity for providing novel starting points in validated binding volumes.

Quantitatively, the SAFR library shows some chemical overlap with the experimental screen, with 23 fragments appearing on both sets (Figure 10C). Crucially, the hotspot analysis (Figure 10D) reveals that the highest density of SAFR predictions occurs inside 2.0 Å of an experimentally verified fragment hotspot, with 88.4% of all fragments in the generated library positioned within this 2.0 Å threshold. This strong concentration in the “success zone” (low distance, high similarity) proves that SAFR-derived fragments consistently recover biologically relevant hotspots, confirming its utility for prospective fragment-based design and its ability to identify high-occupancy binding regions.

### 3.6 Case Study: Identification of Bioisosteric Fragments in the SGLT2 Binding Pocket

Using the crystal structure of the inhibitor Dapagliflozin (PDB code: 8HEZ) as a reference, we focused on a specific fragment-sized substructure occupying a critical subpocket of the sodium/glucose cotransporter 2 (SGLT2), a key therapeutic target for diabetes. To define the pharmacophoric features of this reference, we analyzed the complex using the Protein–Ligand Interaction Fingerprints (ProLIF).^39^ This analysis characterizes a specific network of contacts, identifying critical hydrogen bonds with residues ASN75, HIS80, PHE98, SER287, LYS321, and GLN457, as well as a key π-stacking interaction with HIS80 (Figure 11A). Taking this precise interaction fingerprint, we queried SAFR to find fragments capable of replicating this specific binding mode. We identified 50 distinct fragments that, in their high-confidence predicted poses, were confirmed by ProLIF to replicate this exact same set of interactions (Figure 11B). These newly identified fragments, which function as structural bioisosteres of the original crystallized hit, offer structurally distinct but functionally equivalent starting points for fragment-to-lead or SAR campaigns. Under the same criteria, and using fragments coming solely from X-ray crystal structures, only one fragment would be considered a bioisosteric replacement (PDB entry 8HDH).

**Figure 11:**
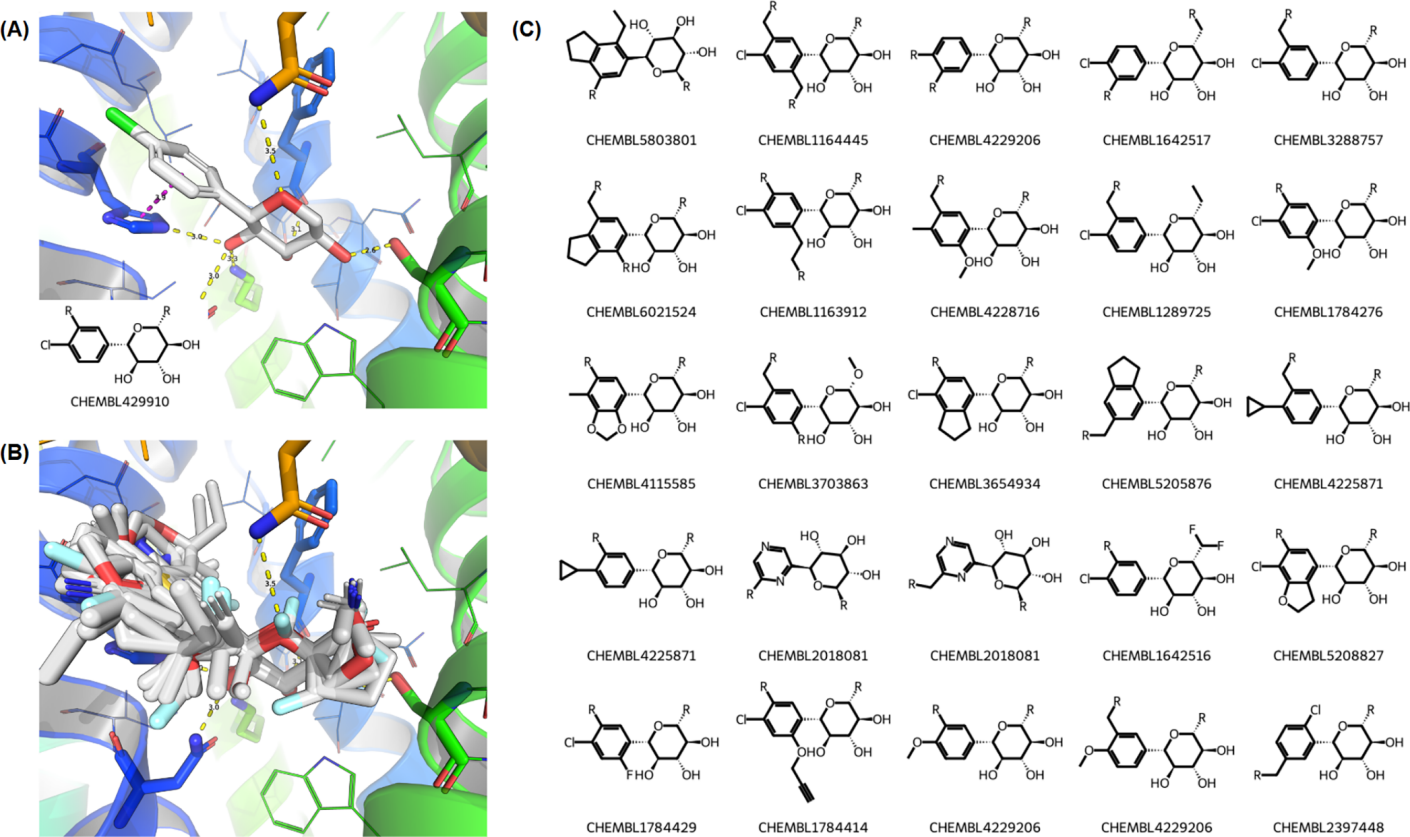
**Case Study: Identification of Novel Bioisosteric Fragments for SGLT2. (A):** The reference binding mode of the crystal fragment from Dapagliflozin (PDB code: 8HEZ), highlighting its key hydrogen bonding and pi-stacking interactions within the SGLT2 binding site. **(B):** An overlay of fragments from our library that are predicted to replicate the same interaction network as the reference fragment. **(C):** 2D structures of 25 of the bioisosteres identified through this interaction-based query where the ChEMBL ID of the parent molecule is shown.

### 3.7 Case Study: Scaffold Hopping for a PIM1 Kinase Inhibitor

Another direct application of SAFR is to enable scaffold hopping campaigns. In this case, SAFR is queried to find novel core structures that can spatially orient the key interacting side chains of a known ligand, maintaining biological activity. Scaffold hopping is a key part of modern medicinal chemistry, used to expand intellectual property, improve physicochemical or ADME properties, and discover entirely new chemical series. To demonstrate this capability, we performed a case study inspired by the work of Rossen et al.^40^ focusing on an inhibitor of the PIM1 kinase (PDB entry 5DWR). The aim is to replace the central fluoropyridine acetamide core of the inhibitor while preserving the 3D orientation of its peripheral, interacting moieties.

Our query identified distinct alternative scaffolds that could substitute the original core based on similar size and compatible attachment vectors, while maintaining the interacting moieties (Figure 12A). Interestingly, these are not naive replacements but are composed of fragments predicted with high confidence to favorably occupy that specific region of the PIM1 binding site. Many of these scaffolds were identified multiple times with a variety of decorations in SAFR. From these candidate scaffold fragments, 10 candidate molecules were generated (Figure 12B). One of them, ChEMBL3676300, derived by cutting and fusing parts of ChEMBL3651966 and ChEMBL4459538, is a confirmed potent inhibitor (*K*_i_ < 1 nM) of PIM1 ^41^ (Figure 12C).

**Figure 12:**
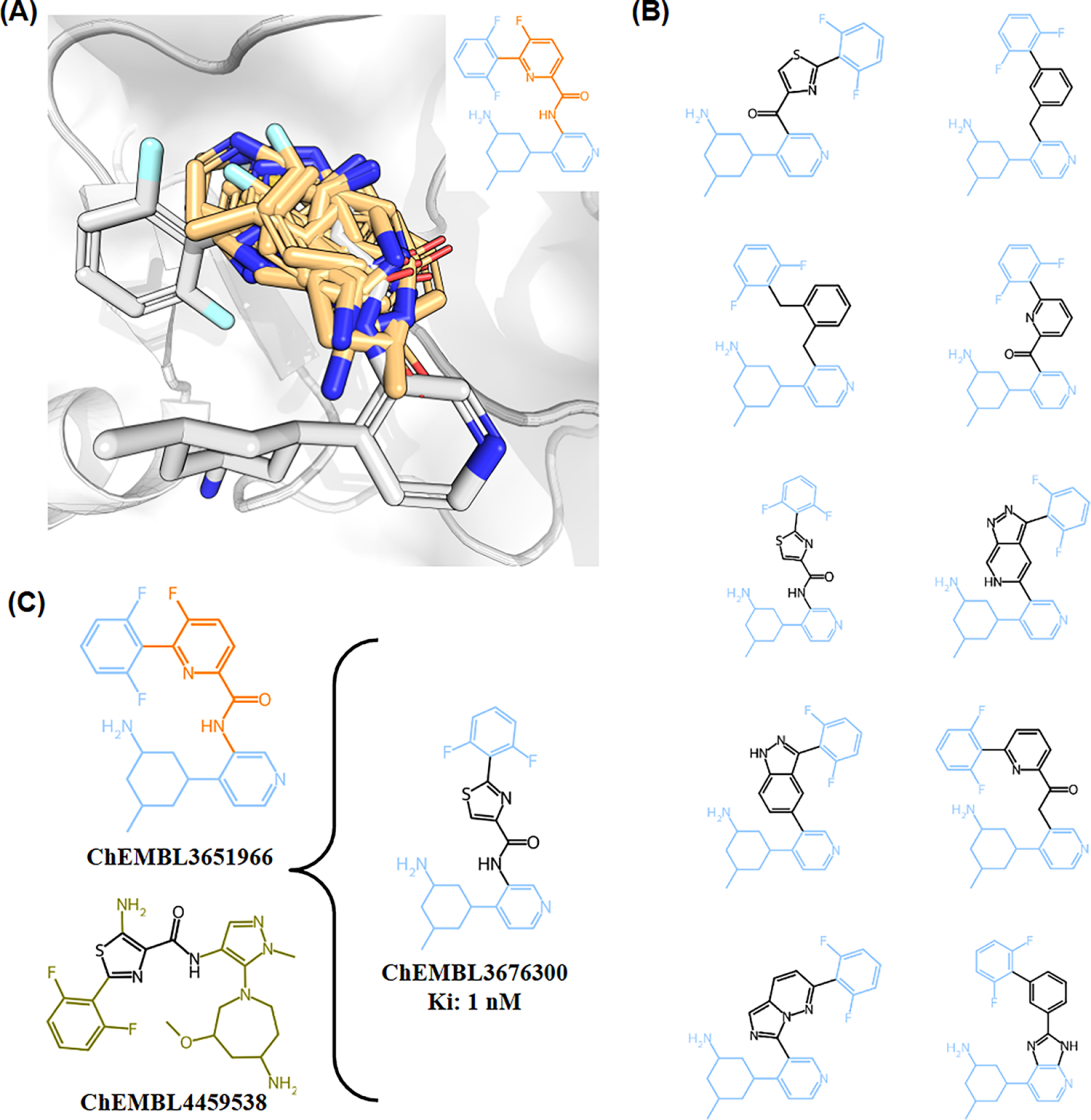
**Scaffold Hopping case study for a PIM1 Kinase Inhibitor.** Results of the scaffold hopping experiment based on the bioactive molecule from PDB 5DWR (ChEMBL3651966). **(A):** 3D overlay of fragments with predicted poses occupying the space that the scaffold occupies in the PIM1 binding pocket. **(B):** Structures of 10 Scaffold-Hopped reconstructed example molecules using fragments with a predicted pose that occupies the same space and has compatible attachment vectors. (C): Structure of one of the reconstructed molecules (ChEMBL3676300) that has been reported in the literature as active against PIM1, to its left ChEMBL4459538 from which the scaffold fragment originated.

### 3.8 Limitations and Future Directions

The strategy presented here has some inherent limitations tied to its knowledge-based nature. The main requisite is the availability of at least one high-quality crystal structure of a similar ligand to be used as a template. Therefore, it cannot be applied to orphan targets distantly related to any protein with a resolved ligand-bound crystal structure or to ligands with entirely distinct structures without any resemblance to known crystallized binders. Furthermore, as highlighted above (Figure 3B), the accuracy is sensitive to significant conformational differences between the template and query ligands. Structurally similar ligands that adopt fundamentally different binding modes remain a challenge.

Additionally, while our local optimization protocol is designed to resolve minor steric clashes, it is not designed to perform the exhaustive conformational sampling needed for highly flexible molecules, which may require more computationally intensive methods like molecular dynamics. Finally, the structural novelty of the predicted fragments is concentrated in therapeutically relevant targets, but it may be less applicable to proteins with few known experimental binders in the PDB.

## 4 Conclusions

Our template-based pose generation provides a robust and scalable solution for converting nonstructural bioactivity data into high-confidence 3D structural hypotheses. It is important to note that SAFR is a knowledge-extractive, structure based resource built to enhance and support machine learning and generative models, rather than serving as a substitute for them. The reliability of this framework was checked through a rigorous, multitiered validation process. This includes large-scale retrospective benchmarking, successful prospective prediction of 14 newly released crystal structures, checks for chemical plausibility, and high concordance with SAIR.

SAFR offers a meticulously curated fragment library, containing over 200,000 structurally annotated fragment-protein pairs. Each fragment in this library has an associated, high-confidence 3D protein binding environment derived from computational modeling. SAFR is specifically enriched with novel fragments and scaffolds targeting all protein families of therapeutical relevance.

SAFR can be a useful resource for next-generation FBDD. It can serve as a starting point for traditional fragment growing, linking, and/or merging campaigns, leveraging the high-confidence 3D context of each fragment but also as a knowledge-guided training set for machine learning models, including 3D-aware generative algorithms. By providing prevalidated, high quality 3D poses, SAFR can be used to design novel molecules fitting within specific binding environments, accelerating the discovery of novel chemical series.

## Supplementary Material



## Data Availability

SAFR alongside the Crystal Fragments set is available at https://zenodo.org/records/18229523. Scripts and files needed to reproduce the analysis performed in this work are available at https://github.com/chemotargets/SAFR.

## Notes

Computational docking and optimization parameters; RMSD values of the 14 ligands that have been released on the PDB after the compilation of our prediction; chemical descriptors for the predicted fragments data set (unique fragment entries); chemical descriptors for the crystal fragments data set (unique fragment entries); sensitivity analysis of confidence score parameter weights; robustness of pose prediction to binding-mode multiplicity; representative failure modes of the template-based pose prediction pipeline; Impact of ligand flexibility on computational runtime; property distributions of the crystal fragments set; top enriched scaffolds present in SAFR uniquely targeting proteins from nuclear receptors, peptidases/proteases, phosphatases, reductases, transcription factors, ion channels, and transporters (PDF)
