# Supplementary material for: SAFR: Enabling Fragment-Based Drug Discovery with a Synthetic Binding Pose Data Set
Source: J Chem Inf Model. 2026 Apr 16;66(8):4848–62. doi: 10.1021/acs.jcim.6c00217 (PMC13126625; doi:10.1021/acs.jcim.6c00217)
Supplement: Supplementary file 1 [file ci6c00217_si_001.pdf]

Supplementary Material

SAFR: Enabling Fragment-Based Drug Discovery with a Synthetic Binding Pose Data Set

Joan Cabot-March[1,2], Xavier Jalencas[1,*], Jordi Mestres[1,2,*]

[1] Chemotargets SL, Parc Cientific de Barcelona, Baldiri Reixac 4 (TR-03), 08028 Barcelona, Catalonia, Spain.
[2] Institut de Quimica Computacional i Catalisi, Facultat de Ciencies, Universitat de Girona, Maria Aurelia Capmany 69, 17003 Girona, Catalonia, Spain.
* Correspondence to: xavier.jalencas@chemotargets.com, jordi.mestres@udg.edu

1. **Listing S1: Computational Docking and Optimization Parameters**

2. **Table S1.** RMSD values of the 14 ligands that have been released on the PDB after the compilation of our prediction.
3. **Table S2.** Chemical descriptors across the Predicted Fragments dataset (unique fragment entries).
4. **Table S3.** Chemical descriptors across the Crystal Fragments dataset (unique fragment entries).
5. **Figure S1. Sensitivity Analysis of Confidence Score Parameter Weights.** Relative variation of the F1-score across the full validation set as a function of percentage perturbations applied to the four constituent weights of the CS function. For each iteration, a single parameter was perturbed while the remaining weights were proportionally rescaled to maintain normalization. Results indicate that the model reaches an optimal performance plateau for *IntSim* and *IntDock* consistent with parameters derived from the CASF-2016 subset. While *3Dsim* shows a slight positive gradient suggesting a minor underestimation in the initial fit, the F1-score remains largely invariant to perturbations in the *2Dsim* weight. This insensitivity suggests that while 2D similarity is essential for initial template selection, it does not act as a primary determinant of 3D pose fidelity.

6. **Figure S2. Impact of ligand flexibility on computational runtime.** Processing time per template plotted as a function of the number of rotatable bonds in the query ligand. The solid red line shows the median runtime over 5 replicates while the shaded region denotes the interquartile range (IQR, representing the 25th to 75th percentiles). To maintain statistical robustness, highly flexible ligands with 20 or more rotatable bonds were grouped into a single 20+ bin. The observed trend illustrates the expected increase in computational cost as the conformational search space expands with additional degrees of freedom. All times reflect execution on a single Intel Xeon Gold 6230R (2.10 GHz) CPU core.
7. **Figure S3. Robustness of pose prediction to binding-mode multiplicity. (A)** Cumulative distribution of median prediction RMSD among 5 runs, grouped by the number of distinct binding pockets per target. **(B)** Boxen plots showing the distribution spread of prediction RMSD across the same pocket count bins. The dotted red lines in both panels denote the 2.0 Å success threshold. The analysis indicates that prediction performance is largely independent of the target's binding-mode multiplicity.
8. **Figure S4. Representative failure modes of the template-based pose prediction pipeline.** In all structural overlays, the experimental crystal pose of the query ligand is shown in white, the reference template pose in purple, and the computationally predicted pose in yellow. **(A) Highly Flexible Ligands:** For molecules with a high number of rotatable bonds (e.g., query 6dne vs. template 6djc), the expanded conformational space may exceed the capabilities of local optimization, trapping the prediction in the template's local minimum. **(B) Inverted Binding Modes:** Highly similar molecules within a congeneric series can adopt flipped or alternative binding orientations (e.g., query 1j07 vs. template 1n5r). The algorithm's reliance on template alignment inherently fails to predict these divergent modes. **(C) High Pocket Plasticity:** Significant structural rearrangements or mutations within the target binding site (e.g., query 3w2r vs. template 3w2o) alter the accessible volume, rendering the rigid spatial constraints of the template incompatible with the query ligand's true binding mode.
9. **Figure S5. Assessment of template reuse and structural bias. (A)** Kernel density estimate and **(B)** empirical cumulative distribution of the number of unique ligands modeled per structural template (logarithmic scale). The dashed red and solid orange lines represent the mean (7.9) and median (3) reuse rates. The data demonstrates that despite a long-tail distribution where a few select templates are highly reused, the overall template usage is broadly distributed, minimizing the propagation of systematic structural bias into the predicted fragment library.
10. **Figure S6. Property Distributions of Crystal Fragment Library. (A):** Grid of distribution plots for nine key properties: Molecular Weight, cLogP, Ring Count, Rotatable Bonds, H-Bond Donors and Acceptors, TPSA, Heavy Atom Count, and Fraction Csp3. The property distribution acts as the baseline for fragment-like properties. **(B):** 2D density heatmap illustrating the distribution of fragments in the chemical space defined by Molecular Weight and cLogP, setting the baseline for the fragment

chemical space. **(C):** Bar plot comparing Rule of Three (Ro3) compliance showing fragment-like character.

11. **Figure S7.** Top enriched scaffolds targeting ion channel proteins from the Predicted Fragments library not appearing in any other target class.
12. **Figure S8.** Top enriched scaffolds targeting nuclear receptors from the Predicted Fragments library not appearing in any other target class.
13. **Figure S9.** Top enriched scaffolds targeting Peptidases/Proteases from the Predicted Fragments library not appearing in any other target class.
14. **Figure S10.** Top enriched scaffolds targeting Phosphatases from the Predicted Fragments library not appearing in any other target class.
15. **Figure S11.** Top enriched scaffolds targeting Reductases from the Predicted Fragments library not appearing in any other target class.
16. **Figure S12.** Top enriched scaffolds targeting Transcription factors from the Predicted Fragments library not appearing in any other target class.
17. **Figure S13.** Top enriched scaffolds targeting Transport proteins from the Predicted Fragments library not appearing in any other target class.

Listing S1: Computational Docking and Optimization Parameters

QuickVina2: QuickVina2 was utilized strictly for scoring optimized poses. The software was executed using default search parameters with the --score_only flag to extract the affinity estimate without further stochastic sampling.

rDock Tethered Docking Configuration: The tethered optimization was performed using a custom parameter file. The cavity was defined using the reference ligand method with a 5.0 Å radius. Key constraints to preserve the initial alignment are:

```
SECTION MAPPER

    SITE_MAPPER RbtLigandSiteMapper

    REF_MOL [TEMPLATE_LIGAND_FILE].sdf

    RADIUS 5.0

    SMALL_SPHERE 1.0

    MIN_VOLUME 100

    MAX_CAVITIES 1

    VOL_INCR 0.0

    GRIDSTEP 0.5

END_SECTION

SECTION CAVITY

     SCORING_FUNCTION RbtCavityGridSF

     WEIGHT 1.0

END_SECTION

SECTION LIGAND

    TRANS_MODE TETHERED

    ROT_MODE TETHERED

    DIHEDRAL_MODE FREE

    MAX_TRANS 0

    MAX_ROT 0

END_SECTION
```

Table S1. RMSD values of the 14 ligands that have been released on the PDB after the compilation of our prediction.

PDB_CID	Uniprot	PDB	Release Date	L_RMSD	P_RMSD
ZVA	Q9NWZ3	8scw	2025-02-12	0.716	0.318
ZVD	Q9NWZ3	8sce	2025-02-12	0.495	0.391
ZVG	Q9NWZ3	8scv	2025-02-12	0.827	0.548
A1IA0	Q99685	9f8a	2025-01-22	0.896	0.286
A1IA1	Q99685	9f8d	2025-01-22	1.606	0.290
A1AD6	P41145	8vve	2025-01-15	1.116	2.280
A1H5L	P34913	8s75	2025-03-12	1.070	0.473
A1H5M	P34913	8s76	2025-03-12	1.200	0.277
A1H3J	P29274	8rw7	2025-01-08	0.547	0.748
A1L0Q	P17931	8z1s	2025-03-05	0.561	0.379
A1L0Q	P17931	8z1t	2025-03-05	1.808	0.255
A1H6U	P14902	9ew0	2025-04-02	1.956	1.648
A1L3A	P08581	9ivb	2025-01-22	1.015	0.655
A1A79	O14744	9dod	2025-02-12	0.732	0.425

Table S2. Chemical descriptors across the Predicted Fragments dataset (unique fragment entries).

Descriptor	Min	Max	Mean	Stddev
Molecular Weight	42.08	299.85	222.55	49.67
Heavy Atom Count	3	23	15.91	3.72
Hetero Atom Count	0	11	4.24	1.58
H-bond Acceptor Count	0	6	3.09	1.40
H-bond Donor Count	0	3	1.17	0.89
Topological Polar Surface Area	0	180.88	52.10	23.50
Wildman-Crippen LogP	-6.04	7.00	1.69	1.39
Rotatable Bond Count	0	5	2.32	1.31
Fraction Csp3	0	1	0.32	0.26
Aliphatic Carbocycle Count	0	4	0.17	0.45
Aliphatic Heterocycle Count	0	4	0.38	0.57
Aliphatic Rings Count	0	4	0.55	0.70
Aromatic Carbocycle Count	0	4	0.74	0.66
Aromatic Heterocycles Count	0	4	0.88	0.82
Aromatic Ring Count	0	4	1.62	0.91
Bridgehead Atom Count	0	4	0.04	0.29
Spiro Atom Count	0	2	0.01	0.12
Ro3 Violations	0	2	0.55	0.57

Table S3. Chemical descriptors across the Crystal Fragments dataset (unique fragment entries).

Descriptor	Min	Max	Mean	Stddev
Molecular Weight	40.06	299.98	214.24	49.62
Heavy Atom Count	3	23	15.15	3.69
Hetero Atom Count	0	12	4.15	1.62
H-bond Acceptor Count	0	6	3.05	1.34
H-bond Donor Count	0	3	1.30	0.92
Topological Polar Surface Area	0	155.05	53.22	24.82
Wildman-Crippen LogP	-4.36	7.09	1.57	1.41
Rotatable Bond Count	0	5	2.37	1.42
Fraction Csp3	0	1	0.36	0.28
Aliphatic Carbocycle Count	0	4	0.14	0.43
Aliphatic Heterocycle Count	0	4	0.41	0.59
Aliphatic Rings Count	0	4	0.56	0.70
Aromatic Carbocycle Count	0	4	0.70	0.66
Aromatic Heterocycles Count	0	4	0.66	0.75
Aromatic Ring Count	0	4	1.36	0.92
Bridgehead Atom Count	0	4	0.03	0.26
Spiro Atom Count	0	2	0.01	0.10
Ro3 Violations	0	2	0.51	0.55

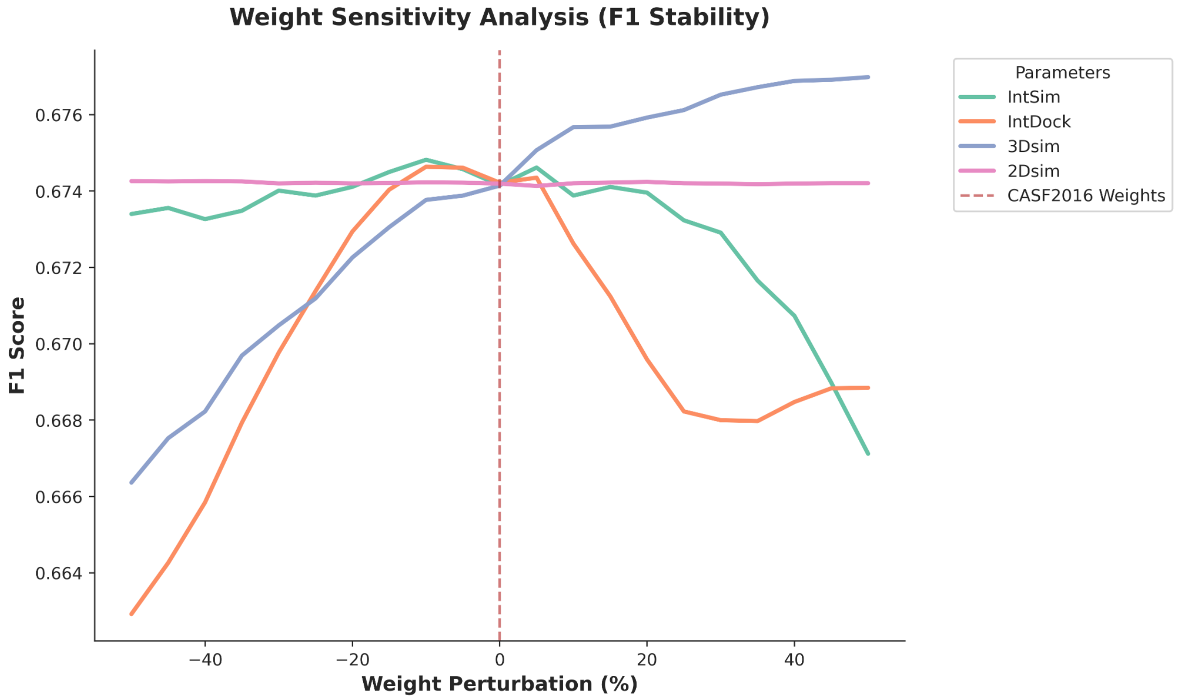

Figure S1. Sensitivity Analysis of Confidence Score Parameter Weights. Relative variation of the F1-score across the full validation set as a function of percentage perturbations applied to the four constituent weights of the CS function. For each iteration, a single parameter was perturbed while the remaining weights were proportionally rescaled to maintain normalization. Results indicate that the model reaches an optimal performance plateau for *IntSim* and *IntDock* consistent with parameters derived from the CASF-2016 subset. While *3Dsim* shows a slight positive gradient suggesting a minor underestimation in the initial fit, the F1-score remains largely invariant to perturbations in the *2Dsim* weight. This insensitivity suggests that while 2D similarity is essential for initial template selection, it does not act as a primary determinant of 3D pose fidelity.

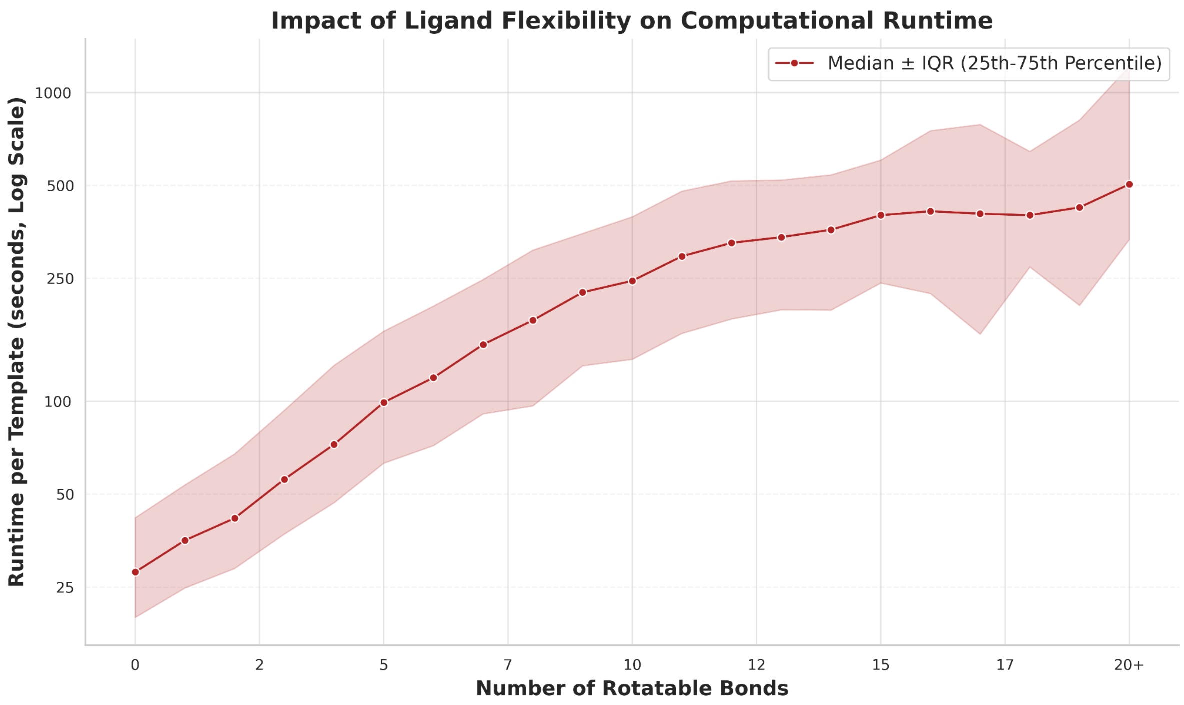

Figure S2. Impact of ligand flexibility on computational runtime. Processing time per template plotted as a function of the number of rotatable bonds in the query ligand. The solid red line shows the median runtime over 5 replicates while the shaded region denotes the interquartile range (IQR, representing the 25th to 75th percentiles). To maintain statistical robustness, highly flexible ligands with 20 or more rotatable bonds were grouped into a single 20+ bin. The observed trend illustrates the expected increase in computational cost as the conformational search space expands with additional degrees of freedom. All times reflect execution on a single Intel Xeon Gold 6230R (2.10 GHz) CPU core.

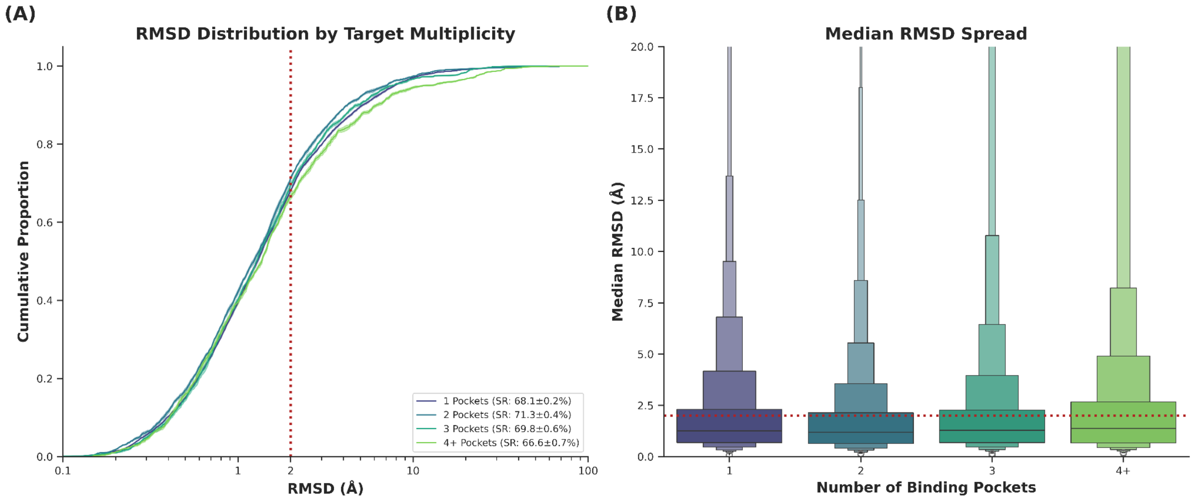

Figure S3. Robustness of pose prediction to binding-mode multiplicity. (A) Cumulative distribution of median prediction RMSD among 5 runs, grouped by the number of distinct binding pockets per target. **(B)** Boxen plots showing the distribution spread of prediction RMSD across the same pocket count bins. The dotted red lines in both panels denote the 2.0 Å success threshold. The analysis indicates that prediction performance is largely independent of the target's binding-mode multiplicity.

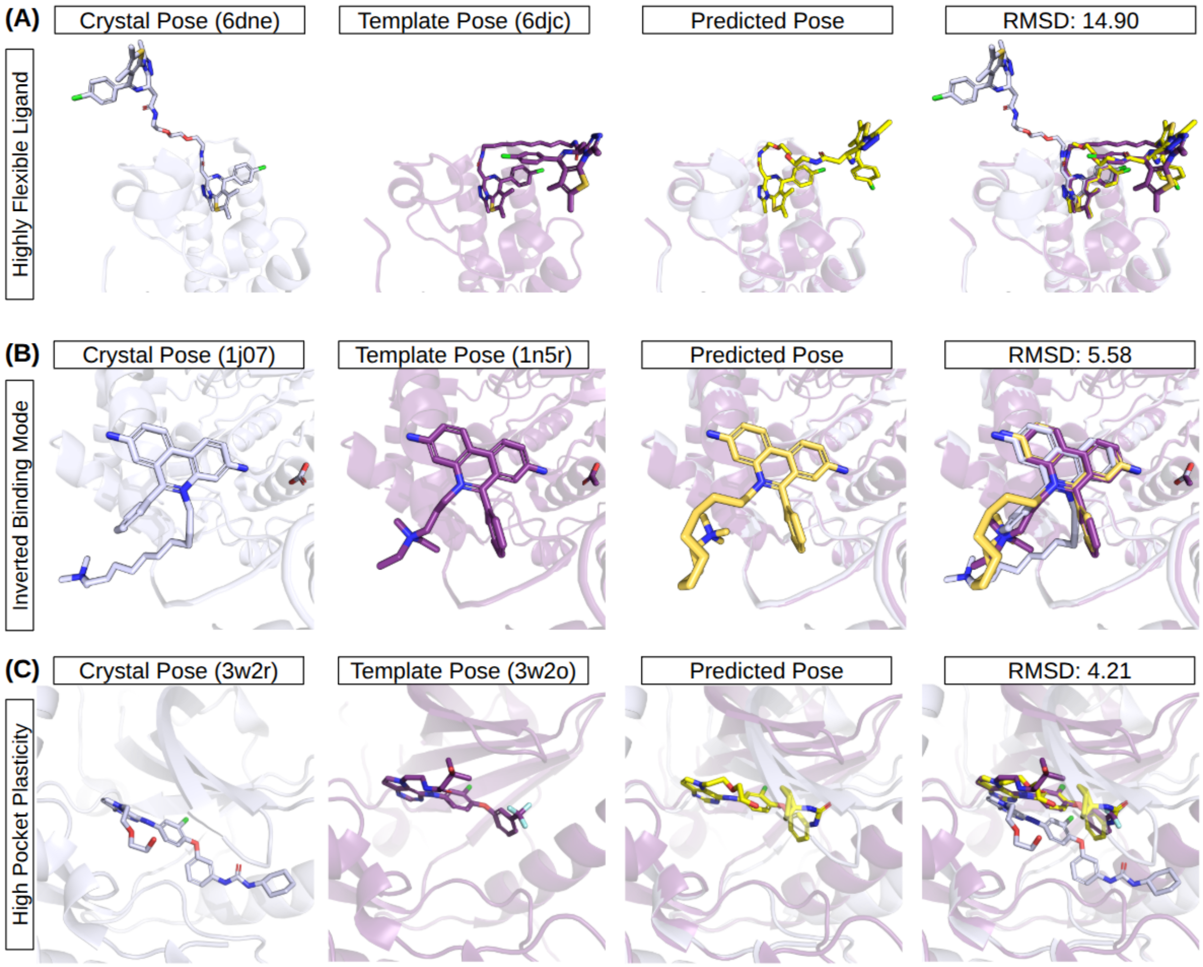

Figure S4. Representative failure modes of the template-based pose prediction pipeline. In all structural overlays, the experimental crystal pose of the query ligand is shown in white, the reference template pose in purple, and the computationally predicted pose in yellow. **(A) Highly Flexible Ligands:** For molecules with a high number of rotatable bonds (e.g., query 6dne vs. template 6djc), the expanded conformational space may exceed the capabilities of local optimization, trapping the prediction in the template's local minimum. **(B) Inverted Binding Modes:** Highly similar molecules within a congeneric series can adopt flipped or alternative binding orientations (e.g., query 1j07 vs. template 1n5r). The algorithm's reliance on template alignment inherently fails to predict these divergent modes. **(C) High Pocket Plasticity:** Significant structural rearrangements or mutations within the target binding site (e.g., query 3w2r vs. template 3w2o) alter the accessible volume, rendering the rigid spatial constraints of the template incompatible with the query ligand's true binding mode.

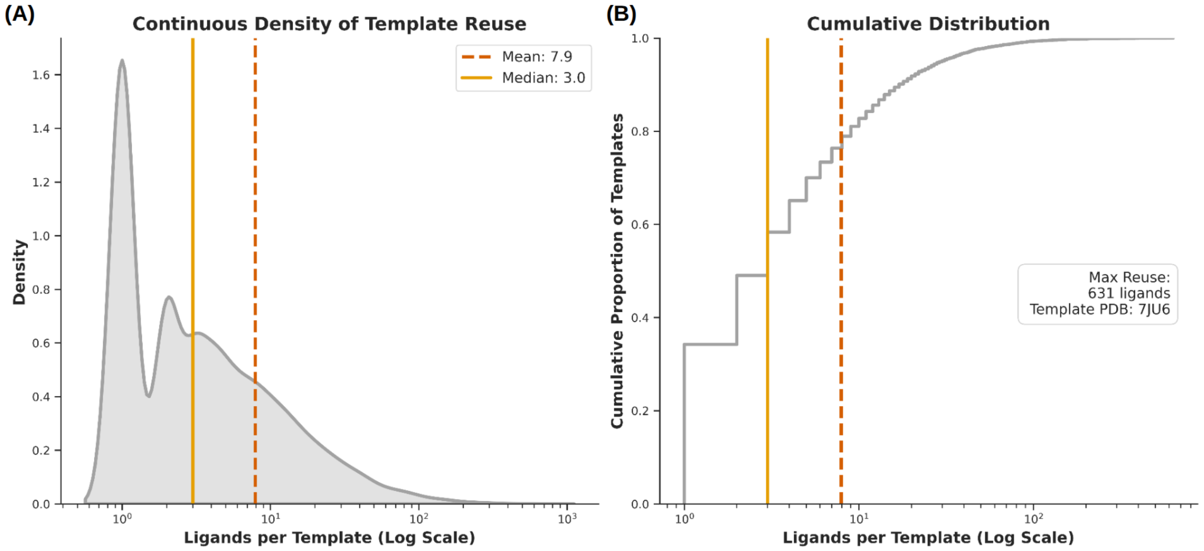

Figure S5. Assessment of template reuse and structural bias. (A) Kernel density estimate and **(B)** empirical cumulative distribution of the number of unique ligands modeled per structural template (logarithmic scale). The dashed red and solid orange lines represent the mean (7.9) and median (3) reuse rates. The data demonstrates that despite a long-tail distribution where a few select templates are highly reused, the overall template usage is broadly distributed, minimizing the propagation of systematic structural bias into the predicted fragment library.

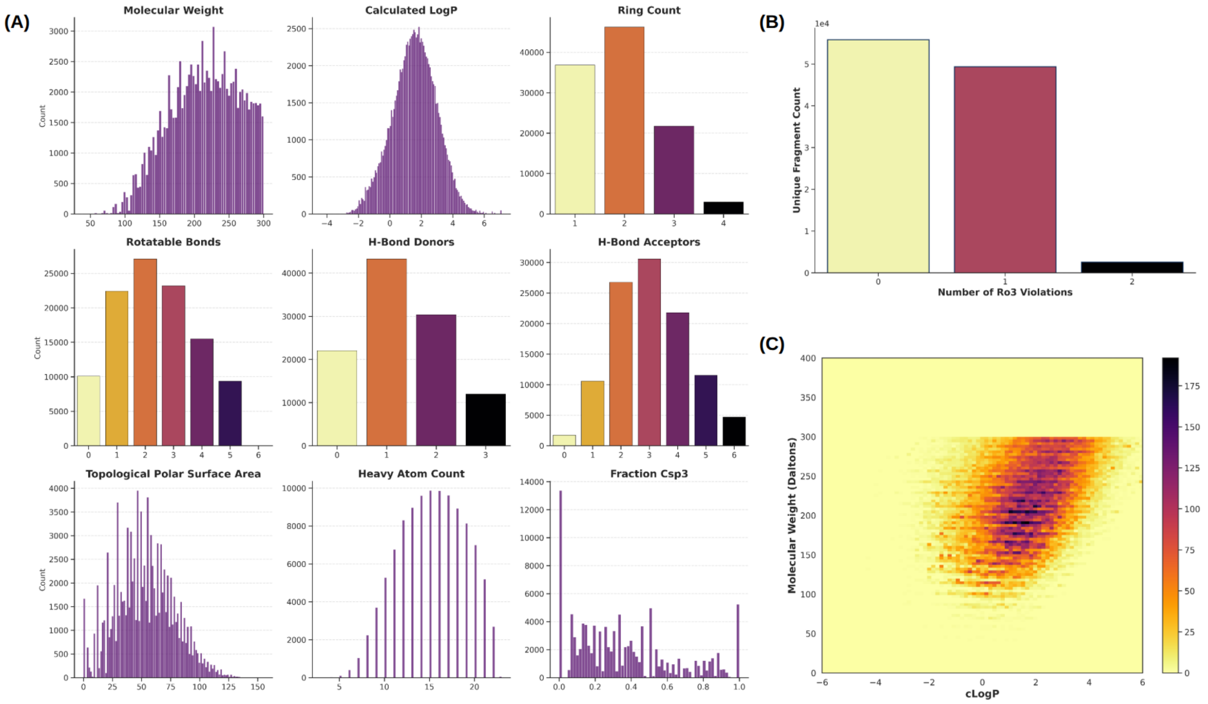

Figure S6 . Property Distributions of Crystal Fragment Library. (A): Grid of distribution plots for nine key properties: Molecular Weight, cLogP, Ring Count, Rotatable Bonds, H-Bond Donors and Acceptors, TPSA, Heavy Atom Count, and Fraction Csp3. The property distribution acts as the baseline for fragment-like properties. (B): 2D density heatmap illustrating the distribution of fragments in the chemical space defined by Molecular Weight and cLogP, setting the baseline for the fragment chemical space. (C): Bar plot comparing Rule of Three (Ro3) compliance showing fragment-like character.

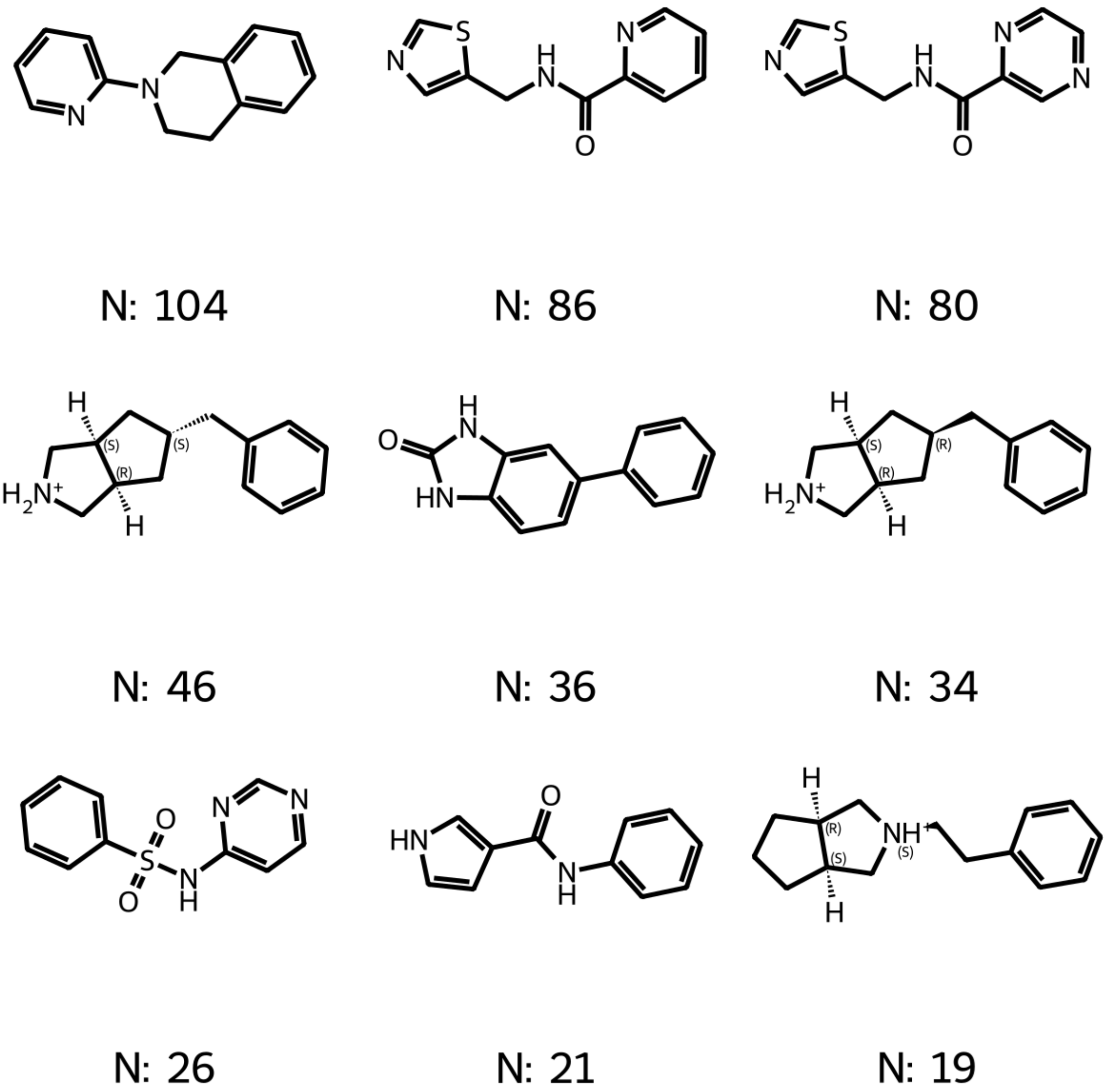

Figure S7 . Top enriched scaffolds targeting ion channel proteins from the Predicted Fragments library not appearing in any other target class.

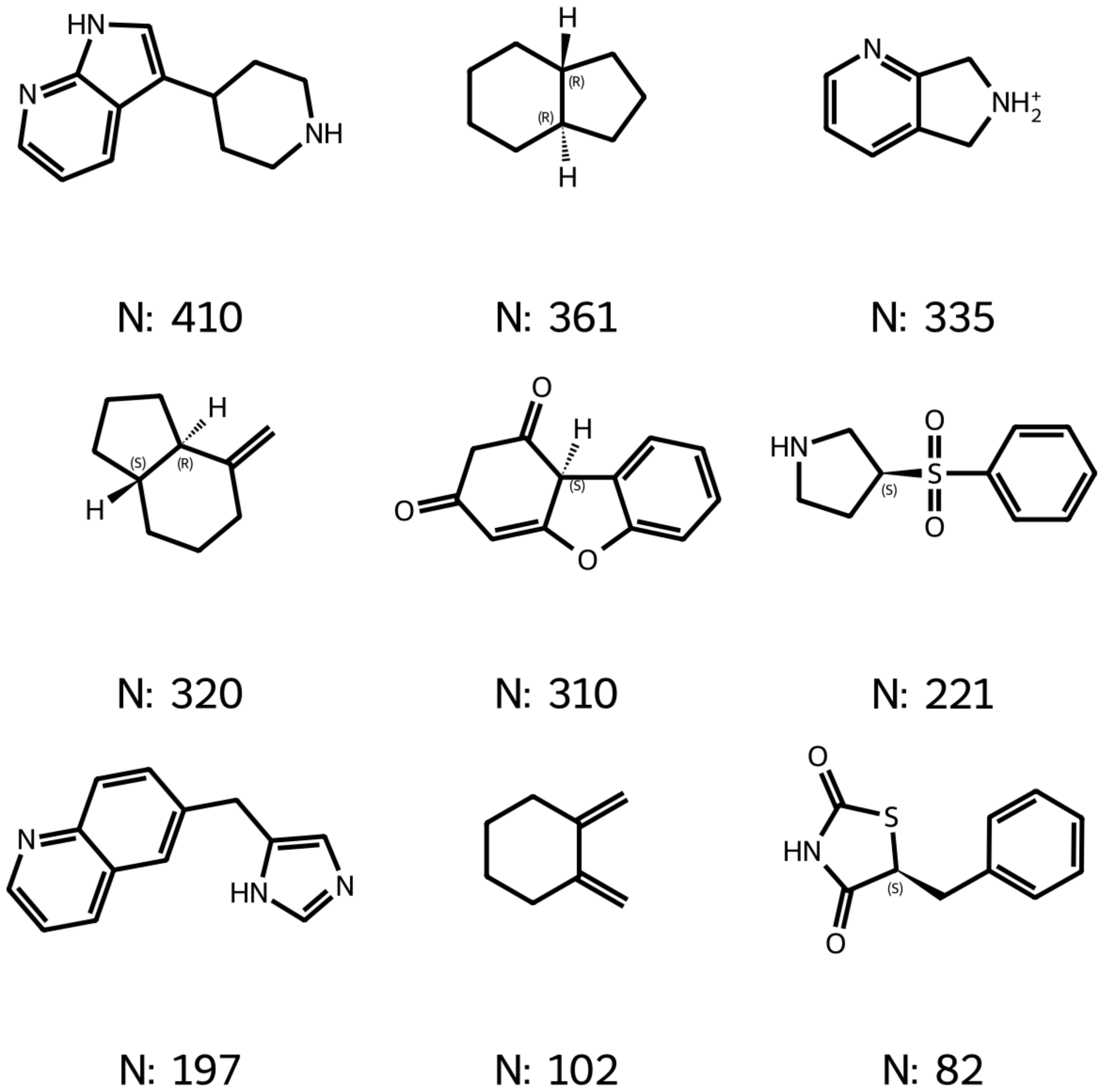

Figure S8. Top enriched scaffolds targeting nuclear receptors from the Predicted Fragments library not appearing in any other target class.

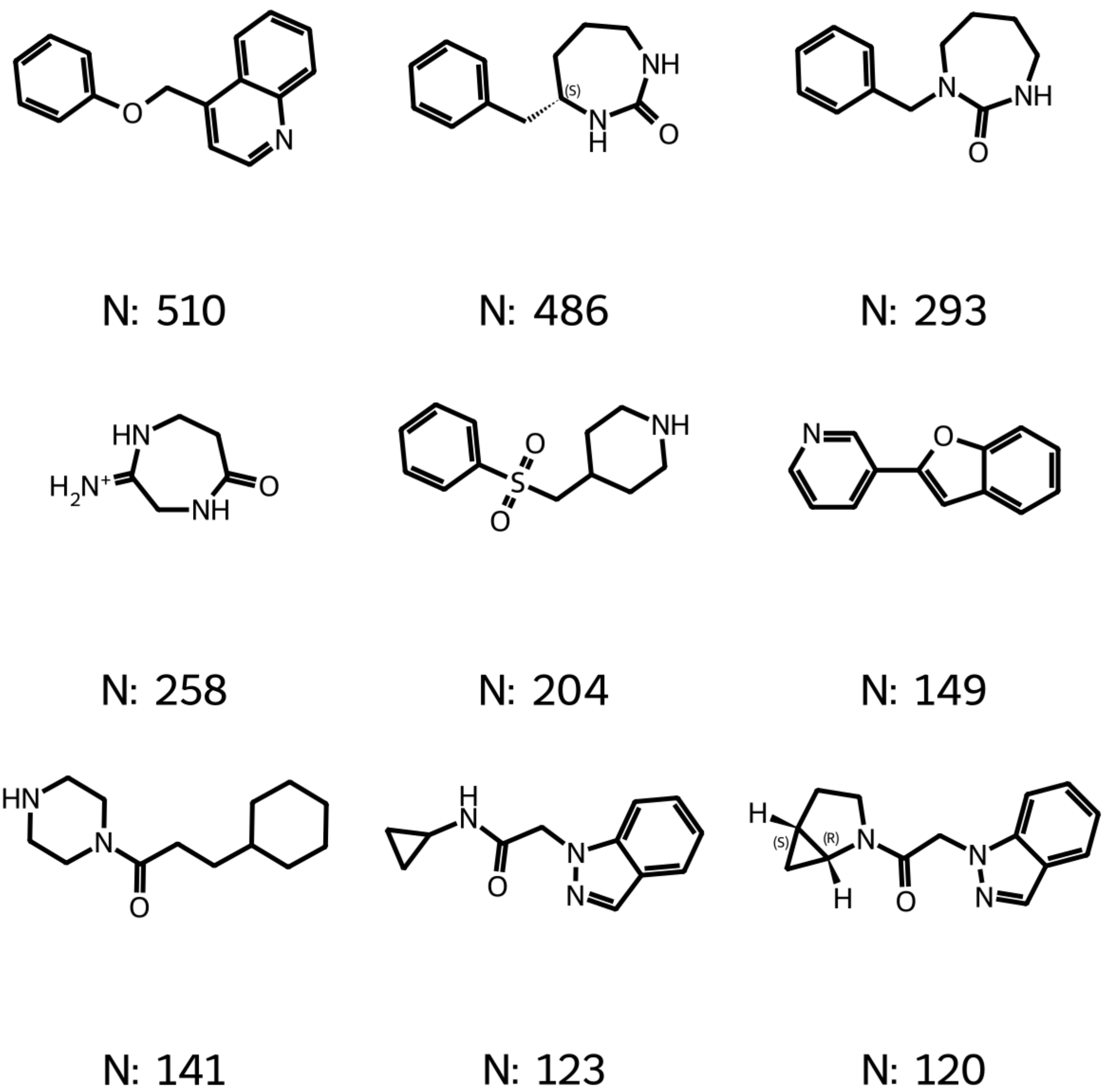

Figure S9. Top enriched scaffolds targeting Peptidases/Proteases from the Predicted Fragments library not appearing in any other target class.

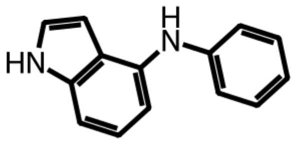

N: 149

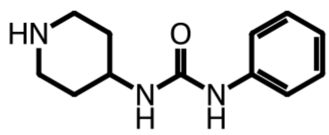

N: 148

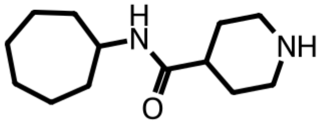

N: 61

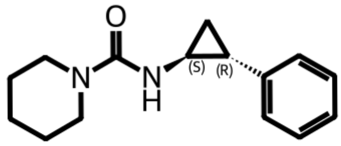

N: 52

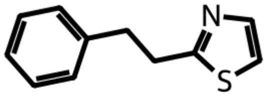

N: 39

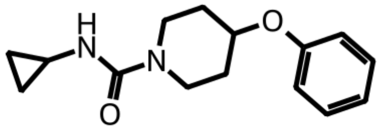

N: 19

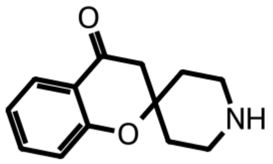

N: 19

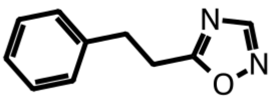

N: 18

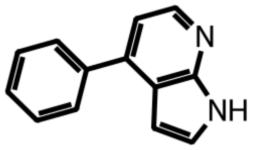

N: 15

Figure S10. Top enriched scaffolds targeting Phosphatases from the Predicted Fragments library not appearing in any other target class.

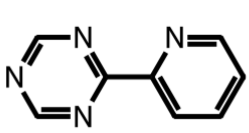

N: 781

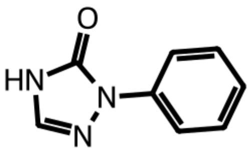

N: 775

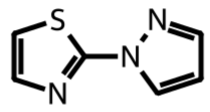

N: 242

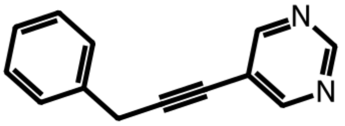

N: 210

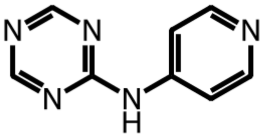

N: 178

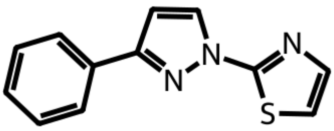

N: 142

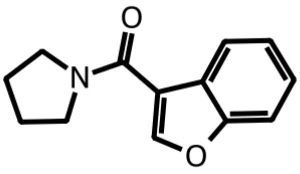

N: 132

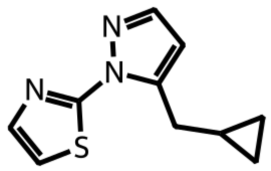

N: 102

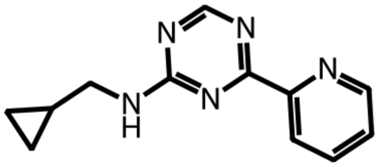

N: 91

Figure S11 . Top enriched scaffolds targeting Reductases from the Predicted Fragments library not appearing in any other target class.

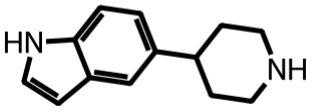

N: 515

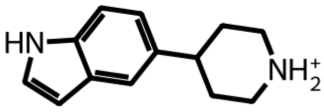

N: 429

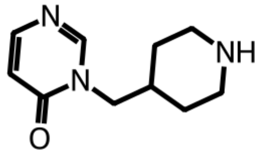

N: 128

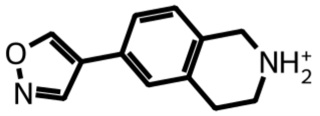

N: 106

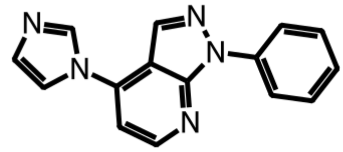

N: 98

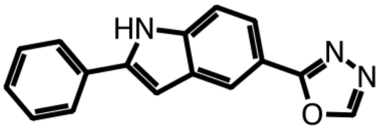

N: 70

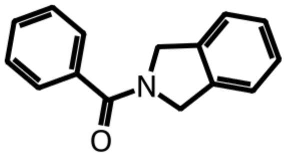

N: 68

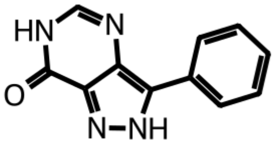

N: 65

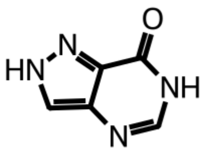

N: 56

Figure S12 . Top enriched scaffolds targeting Transcription factors from the Predicted Fragments library not appearing in any other target class.

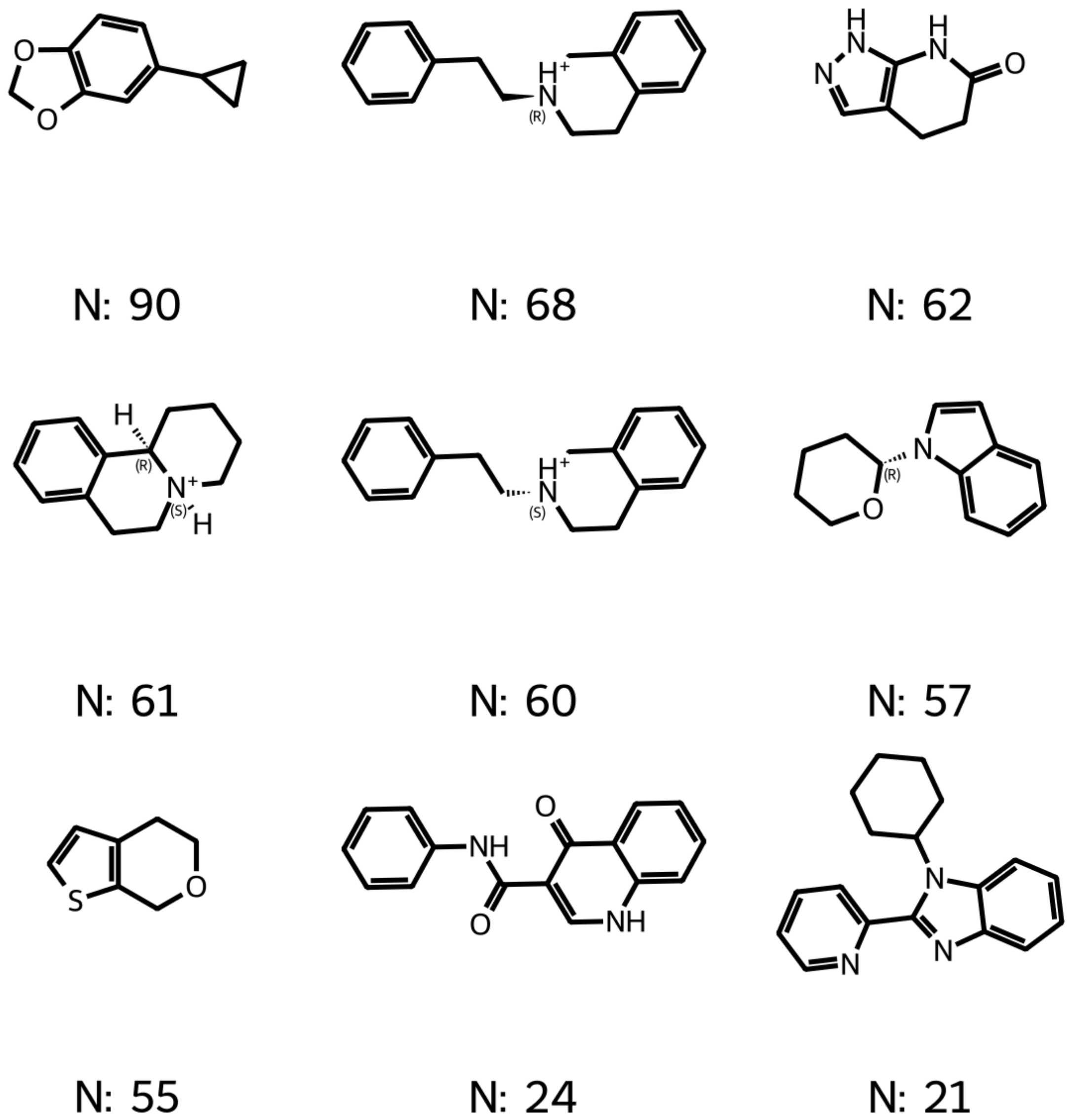

Figure S13. Top enriched scaffolds targeting Transport proteins from the Predicted Fragments library not appearing in any other target class.
